# Expert participation in the health technology assessment process: a scoping review

**DOI:** 10.1590/0034-7167-2025-0157

**Published:** 2026-03-16

**Authors:** Sandra Maria dos Santos Figueiredo, Fernanda de Nazaré Almeida Costa, Marcia Helena Machado Nascimento, Cristiane Cardoso de Paula, Liane Bahú Machado, Eliana Rosa da Fonseca, Elizabeth Teixeira

**Affiliations:** I Centro Universitário do Estado do Pará. Belém, Pará, Brazil; II Universidade do Estado do Pará. Belém, Pará, Brazil; III Universidade Federal de Santa Maria. Santa Maria, Rio Grande do Sul, Brazil; IV Universidade Federal do Rio de Janeiro. Rio de Janeiro, Rio de Janeiro, Brazil

**Keywords:** Technology Assessment, Biomedical, Peer Review, Biomedical Technology, Equipment and Supplies, Methods, Evaluación de la Tecnología Biomédica, Revisión por Pares, Tecnología Biomédica, Equipos y Suministros, Métodos

## Abstract

**Objectives:**

to map evidence on specialist participation in the health technology assessment process.

**Methods:**

a scoping review was conducted using the JBI method, searching the MEDLINE/PubMed, Scopus, EMBASE/Elsevier, WoS/Clarivate, CINAHL, ASP/EBSCO, APA PsycINFO, ERIC, LILACS/BVS, SciELO, and INAHTA databases. Original articles published between 2013 and 2023 were considered. Data were extracted from 89 studies and analyzed using descriptive statistics.

**Results:**

between three and 15 specialists (64% of studies) participated. The property attribute was assessed for digital and non-digital devices, management systems, and equipment (93.1%, 94.2%, 60%, 33.3%, respectively), the cost-effectiveness attribute, for medications (60%), and the utility attribute, for medical-surgical procedures (31.6%). Participation was online, using digital and non-digital devices (53% and 52%, respectively), in-person, using devices (77.7%), and mixed, for the other types.

**Conclusions:**

it is necessary to structure the assessment according to the specific characteristics of the technology type, providing insights for implementing innovations in this sector.

## RESUMEN

**Objetivos:**

mapear la evidencia sobre la participación de expertos en el proceso de evaluación de tecnologías sanitarias.

**Métodos:**

se realizó una revisión exploratoria utilizando el método JBI, con búsquedas en las bases de datos MEDLINE/PubMed, Scopus, EMBASE/Elsevier, WoS/Clarivate, CINAHL, ASP/EBSCO, APA PsycINFO, ERIC, LILACS/BVS, SciELO e INAHTA. Se consideraron artículos originales publicados entre 2013 y 2023. Se extrajeron datos de 89 estudios y se analizaron mediante estadística descriptiva.

**Resultados:**

participaron entre tres y 15 expertos (64% de los estudios). La evaluación del atributo de propiedad se realizó en dispositivos digitales y no digitales, sistemas de gestión y equipos (93,1%, 94,2%, 60%, 33,3%, respectivamente), el atributo de coste-efectividad en medicamentos (60%) y el atributo de utilidad en procedimientos médico-quirúrgicos (31,6%). La participación fue en línea, utilizando dispositivos digitales y no digitales (53% y 52%, respectivamente), presencial, utilizando dispositivos (77,7%), y mixta, para los demás tipos.

**Conclusiones:**

es necesario estructurar la evaluación según las características específicas del tipo de tecnología, aportando perspectivas para la implementación de innovaciones en este sector.

## INTRODUCTION

Health technologies are classified according to the nature of the material: medication (MED); equipment and supplies (EQS); medical-surgical procedure (MSP); management system (MS); and digital device (DD)^(1)^. MED and EQS are considered biomedical technologies, which are those that interact directly with users; MSP constitutes medical technologies, which are essential for the quality in the application of biomedical technologies, such as surgical techniques, technical standards, among others; MS refers to organizational support systems, which encompass a technical and administrative support structure, and information systems and healthcare delivery organization, which, together with medical technologies, make up healthcare technologies; and DD is intervention accessible through mobile devices. There are other classifications regarding the purpose of the technology and stage of diffusion^(1)^, including typologies used by Brazilian nursing.

The life cycles of a technology are innovation, initial diffusion, incorporation, large-scale use, and abandonment. The technological innovation process (first phase of the cycle) begins with the invention of a new product, process, or practice and ends with its first practical use (initial diffusion - second phase of the cycle)^(2)^. Assessment occurs both during the product development or creation phase (prior to registration) and during the product’s incorporation and use phase (post-application).

However, the assessment process is evolving toward more constructive approaches from the technology’s inception through its obsolescence. Therefore, new concepts are being applied, such as scientific advice, early dialogue, post-introduction monitoring of health technologies, adaptation, reassessment, and divestment, among others^(2)^. The adoption of a completer and more holistic “life cycle approach” concept is under discussion, which can promote stakeholder engagement and robust evidence generation^(3)^.

Health technology assessment (HTA) is a multidisciplinary process that uses explicit methods to determine the value of a health technology at different points in its life cycle. The goal is to inform decision-making to promote an equitable, efficient, and high-quality health system^(4)^.

Most HTAs focus on clinical medicine and pharmaceuticals, resulting in a dearth of assessments focused on public health interventions^(5)^. The assessment of complex public health interventions faces challenges, including evidence heterogeneity, which stems from the methodological diversity of studies. HTA contributes to this legitimacy with content and procedures. Content represents the robust and reliable evidence that will be used for informed decision-making, while procedures relate to the principles of good governance that ultimately build trust^(6)^.

It is important to note that, at all stages and life cycles of a technology, different actors can participate in the assessment process. Each stage of the HTA process uses different sources of information and involves different groups of people^(7)^. A specialist in the HTA process refers to external individuals, not regular members of the HTA team, who hold specialized knowledge or expertise in a relevant domain. The term “specialist opinion” encompasses contributions these individuals can offer to the HTA process, including quantitative and qualitative data, as well as experience and value assessments^(8)^.

To assess the need and feasibility of mapping methodological experiences and gaps in the field of specialist participation in the HTA process, a preliminary search for review protocols and/or reports was conducted. Considering information sources such as Medical Literature Analysis and Retrieval System Online (MEDLINE)/PubMed, Cochrane Database of Systematic Reviews, JBI Evidence Synthesis, International Prospective Register of Systematic Reviews, and Open Science Framework (OSF), 2,197 references were identified. This pre-analysis identified a guideline review that reinforces the importance of considering the use of specialist opinion in HTA, mentioning that, despite existing recommendations, there is a scarcity of reports on specialist elicitation methods^(8)^. Specialist participation occurs through various modalities, which can be systematically mapped in the literature, favoring methodological decision-making in studies involving HTA specialists.

## OBJECTIVES

To map evidence on specialist participation in the HTA process.

## METHODS

### Ethical aspects

Since this was a secondary study, extracting data from the literature, no ethical review was required. It is important to note that copyright was respected, with proper citation and referencing of primary studies.

### Study design

This is a scoping review conducted using the JBI method^(9)^. For writing quality and transparency, the Preferred Reporting Items for Systematic reviews and Meta-Analyses extension for Scoping Reviews^(10)^ was applied. The protocol was registered with OSF (https://doi.org/10.17605/OSF.IO/RE8TW).

### Search strategy and data source

The PCC mnemonic was adopted to structure the review question and map terms to compose the search strategy for the information sources: Population (P): specialists; Concept (C): HTA; Context (C): any healthcare setting. The review question was: what are the characteristics of specialist participation in the HTA process?

The search strategy was structured by a librarian with expertise in review studies in the health field. To develop the strategy, a term mapping was developed using, first, the descriptors and words used in the preliminary search, which was expanded with a pre-analysis of identified documents, including other terms contained in titles, abstracts, and indexing terms. Thus, the following search strategy applied to MEDLINE/PubMed was structured: ((Specialist*[tiab] OR expert*[tiab] OR “Consensus”[mh] OR Consensus[tiab] OR “Consensus Development”[tiab] OR “expert committee”[tiab] OR “expert panel”[tiab] OR “expert elicitation”[tiab] OR “effectiveness assessment”[tiab] OR “expert views”[tiab] OR “expert committee”[tiab] OR “expert panel”[tiab] OR “expert elicitation”[tiab] OR “Delphi Technique”[mh] OR “Delphi Method”[tiab] OR “Delphi Studies”[tiab] OR “Delphi Study”[tiab] OR Delphi Technic*[tiab] OR Delphi Technique*[tiab])) AND (“Technology Assessment, Biomedical”[mh] OR “Biomedical Technology Assessment”[tiab] OR (“Biomedical Technology”[mh] AND assessment*[tiab]) OR Health Technology Assessment*[tiab] OR HTA[tiab] OR ((Health Technolog*[tiab] OR Health Care Technolog*[tiab] OR Health Technolog*[tiab] OR Medical Technolog*[tiab] OR care technolog*[tiab] OR caring technolog*[tiab]) AND (assessment*[tiab])))) AND (((2013:2022[pdat]) AND (English[Filter] OR Portuguese[Filter] OR Spanish[Filter]))).) AND (English[Filter] OR Portuguese[Filter] OR Spanish[Filter]))). This strategy was adapted for each information source.

Bibliographic search was conducted on June 19, 2023, using the following sources: MEDLINE (PubMed); Scopus (Elsevier); Excerpta Medica dataBASE (EMBASE (Elsevier)); *Literatura Latino-Americana and do Caribe em Ciências da Saúde; Índice Bibliográfico Español en Ciencias de la Salud; Base de Dados de Enfermagem*; Western Pacific Region Index Medicus; MULTIMEDIA; *Centro Nacional de Información de Ciencias Médicas de Cuba*; World Health Organization Library & Information Networks for Knowledge*; Literatura Peruana en Ciencias de la Salud*; IRIS PAHO; *Base Internacional de Guias GRADE*; PREPRINT-medRxiv; ARGMSAL; *Bibliografía Nacional en Ciencias de la Salud Argentina; Localizador de Informação em Saúde; Políticas Informadas por Evidências*; ColecionaSUS; *Secretaria de Estado da Saúde de São Paulo; Campusvirtualsp_brasil; Bibliografia Brazileira de Odontologia*; IndexPsi; African Index Medicus; DESASTRES; PREPRINT-Scientific Electronic Library Online (SciELO) (Virtual Health Library (VHL)); Education Resources Information Center (ERIC) Core Collection (Clarivate Analytics); Cumulative Index to Nursing and Allied Health Literature and Academic Search Premier (EBSCO); American Psychological Association (APA PsycINFO); International HTA Database; and SciELO.

After the search, duplicates were checked in the EndNote 20/2020 reference manager (Clarivate Analytics, PA, USA).

### Eligibility criteria

The incorporated studies were analyzed for inclusion or exclusion based on the PCC, and were defined as follows: Population (P) - Includes studies in which specialists participated in some stage of the HTA process: people external to the research team with knowledge or experience in a relevant area and who participated individually, on a panel or in a committee, at different moments of the process, i.e., in some of the HTA stages. Concept (C) - Includes HTA studies that determine the value of a health technology at different points in its life cycle using different methods, designs, techniques, and instruments, considering clinical, social, ethical, and economic aspects. These studies may include evidence of internal properties, safety, efficacy, patient-reported outcomes, real-world effectiveness, cost, and cost-effectiveness, as well as social, legal, ethical, and political impacts. Context (C) - Includes studies conducted in any healthcare setting, without specifying the location where HTA was developed, to avoid limiting the search and selection process and to enable the mapping of this characteristic in the literature.

Original articles published in English, Portuguese, or Spanish since 2013 were included, taking into account the Pan American Health Organization’s^(11)^ framework to encourage the establishment of decision-making processes for the incorporation of HTA-based health technologies. Review studies, editorials, letters to the editor, translation and cultural adaptation studies of instruments, and studies in which participation was aimed at assessing strategies, parameters, or guidelines for HTA were excluded.

### Study selection, data extraction, analysis, and presentation of results

As a quality criterion, to minimize the chance of error, the selection and extraction stages were developed independently. When consensus was not reached, a third reviewer was consulted to make the decision. The reviewers were trained on the eligibility criteria. Both the selection checklist (inclusion and exclusion criteria) and the extraction tool were piloted.

The selection stage was developed using the Rayyan^®^ platform (http://rayyan.qcri.org), developed by the Qatar Computing Research Institute. The first stage of selection consisted of reading titles and abstracts between October 2023 and January 2024. Articles that met the eligibility criteria proceeded to the second stage and were read in full for the second selection phase, which took place between February and April 2024. The Rayyan^®^ platform used Blind ON or Blind OFF applications to manage blinding and conflict checking.

Data extraction was developed, according to the JBI instrument, in a Microsoft Excel^®^ spreadsheet structured with two tabs, consistent with the JBI method, which refers to characterization (tab 1: year; country; objective; technology’s target audience) and response to the mapping scope (tab 2: HTA characteristics, such as technology typology, assessed attribute, design context and data collection technique, and specialist characteristics, such as area, sample and selection).

The typology initially started from a theoretical classification according to the World Health Organization (WHO)^(1)^: MED; EQS; MSP; MS; and DD. The DD typology emerged from the mapping and was included later, characterizing itself as an empirical classification.

In the analysis, the data were verified using descriptive statistics to present the study characterization results: temporal trend; geographic distribution; and target population. The results were presented in tabular form and accompanied by a narrative summary, as well as temporal and geographic graphs, which were created for comparison in Napkin and Photoshop.

## RESULTS

The following references were identified, totaling 8,938 studies: 1,172 in VHL; 733 in EBSCO; seven in ERIC; 1,094 in EMBASE; 570 in the HTA Database; 2,015 in APA PsycINFO; 1,056 in Web of Science; 1,869 in Scopus; 100 in SciELO; and 2,015 in PubMed.

**Figure 1:**
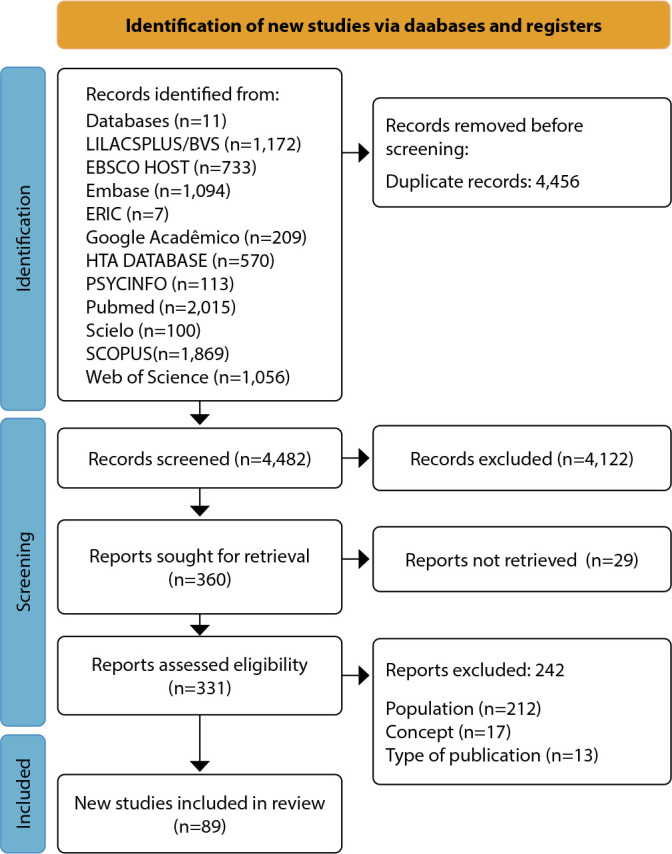
Preferred Reporting Items for Systematic reviews and Meta-Analyses extension for Scoping Reviews 2020 flowchart for identifying and including studies

Of these, 4,456 were excluded due to duplication, and 4,482 were selected for title and abstract reading. Subsequently, 4,122 were excluded because they did not address the research question. Thus, 360 were selected and 29 were eliminated because they were not accessible in full. Moreover, 331 were read, 242 of which were excluded. Finally, 89 studies were included in this review (Figure 1).

A mapping of the characteristics of studies on specialists’ participation in the HTA process was developed regarding geographic location, time, sample, area of knowledge, typology of the technology assessed and technology’s target audience (Chart 1).

**Chart 1:** Characterization of primary studies included in the review in chronological order of publication, Belém, Pará, Brazil, 2024

Country/year	Objective	Sample/area	Type	Audience
Iran 2013^(12)^	Compare patient factors, safety, and organizational considerations for HTA mixture compared to root canal therapy.	6 H and OA	MSP	AOA
United Kingdom 2013^(13)^	Assess treatment for venous leg ulcers associated with bandages and compression garments.	NI H	MSP	AOA
United Kingdom 2013^(14)^	Investigate similarities and differences between Hydration Response Technology and negative pressure wound therapy regarding wound bed preparation and develop recommendations for their use.	23 H	MSP	AOA
Italy 2013^(15)^	Assess the overall technological status of mitral clips in mitral regurgitation treatment, with particular reference to traditional methods, and contextualize the analyses within the hospital structure, identifying critical issues and improvements.	NI H	NDD	AOA
Croatia 2013^(16)^	Develop, implement, and assess student-written patient therapy letters as a tool in family medicine education.	3 H	NDD	STU
Spain 2013^(17)^	Agree among specialists on the content of new maternal education based on women’s health needs and analyze theoretical models and maternal educational strategies to meet these needs.	11 H	NDD	P
Brazil 2013^(18)^	Describe the process of assessing educational technology for *cordel* literature (a popular, inexpensive form of Brazilian narrative poetry) regarding content aspects of breastfeeding.	6 H	NDD	AOA
Argentina 2013^(19)^	Assess the cost-effectiveness of Oxytocin in Uniject in Latin America and the Caribbean.	8 H and OA	MED	AOA
Cuba 2014^(20)^	Discuss a computer tool that helps determine health actions related to hypertension in Primary Health Care through online analytical processing and case-based reasoning.	9 H	MS	AOA
Germany 2014^(21)^	Discuss the perspectives, demands, and safety and risk concerns associated with lab-on-a-chip systems.	30 H	MS	AOA
Brazil 2014^(22)^	Develop and reach consensus on assistive technology for daily clinical assessment of postoperative patients, based on theoretical support and adapting to hospital dynamics.	9 H	MSP	AOA
United Kingdom 2014^(23)^	Determine the most cost-effective diagnostic testing strategy for heavy menstrual bleeding and postmenopausal bleeding diagnosis and treatment.	14 H	MSP	AOA
United Kingdom 2014^(24)^	Identify and describe the selective decontamination of the digestive tract intervention, the evidence base, the acceptability of future research, and the feasibility of new randomized clinical trials.	36 H	MSP	AOA
Peru 2015^(25)^	Develop an online healthcare information technology service and help inform the medical equipment assessment and procurement processes, according to the market and the needs of healthcare institutions.	NI H	MS	P
Brazil 2015^(26)^	Assess the functional performance and technical quality of the Electronic Nursing Process Documentation System.	37 H and OA	MS	P
United Kingdom 2015^(27)^	To explore and promote improvements in evidence generation for Health Technology Assessment in the field of medicines, considering the needs of different stakeholders throughout the entire lifecycle of health technologies	75 H	MED	P
Spain 2015^(28)^	Identify user needs for a new medical technology for managing and monitoring patients with Parkinson’s disease and use these user needs to conduct a preliminary assessment of a specific system called PERFORM, including a case study.	16 H and OA	MS	AOA
Brazil 2015^(29)^	Present the Diabetes Food Control app to assess dietary markers in diabetics, based on a validated questionnaire.	NI H	DD	AOA
Brazil 2015^(30)^	Present Alz Memory, a game developed to stimulate the memory of Alzheimer’s patients, aiming to minimize the effects of the disease.	10 OA	DD	AOA
Argentina 2015^(31)^	Assess the cost-effectiveness of oxytocin in Latin America and the Caribbean.	30 H	MED	AOA
Brazil 2016^(32)^	Assess the usability of a digital educational prototype on a new method for minimally invasive intracranial pressure monitoring for nurses and physicians.	4 OA	DD	P
Spain 2016^(33)^	Understand the criteria of hypertension specialists regarding fixed-dose triple combinations and explore possible differences in criteria regarding their use among physicians in three specialties involved in blood pressure control: internal medicine, cardiology, and nephrology.	158 H	MSP	AOA
Brazil 2016^(34)^	Develop and preliminarily assess a mobile application to assist voice professionals in managing their vocal health.	3 H	DD	P
United States of America 2016^(35)^	Determine whether the application of the Headroom method combined with return-on-investment analysis allows estimating the potential commercial viability of a therapeutic device and five diagnostic devices.	NI H	MSP	P
Netherlands 2016^(36)^	Assess the short-term cost-effectiveness of four complementary diagnostic tests in patients with early inflammatory arthritis at risk for rheumatoid arthritis.	NI OA	MSP	AOA
India 2016^(37)^	Report the implementation process of a tablet-based Mother and Child Tracking System for maternal and child health tracking in Bihar.	NI H and OA	MS	P
Finland 2016^(38)^	Analyze the health technology adoption phase.	NI H	MSP	P
Poland 2016^(39)^	Assess the costs and consequences of using an innovative medical technology, the misoprostol vaginal insert, for labor induction instead of alternative technologies used as standard of care.	5 H	MSP	AOA
Colombia 2017^(40)^	Validate the impact of the biomedical technology management audit tool.	12 H and OA	MS	P
Sweden 2017^(41)^	How accurate is remote image-based diagnosis of burns commonly presented in emergency centers in the Western Cape when viewed on a handheld device? Do remote assessments have comparable accuracy when performed on handheld devices compared to a computer screen?	26 H	MSP	P
Spain 2017^(42)^	Describe the variety of HTA agreements, methods, and processes in the adoption and use of a controversial preventive intervention in selected countries in Central, Eastern, and Southeastern Europe, and Latin America and the Caribbean, as well as vaccine implementation in lower-middle-income countries, and provide recommendations for those countries.	3 H and OA	MSP	NBC and ADO
China 2017^(43)^	Develop a new mobile application for information and social and emotional support for women with breast cancer undergoing chemotherapy to promote self-efficacy and social support, improving symptom management strategies.	8 H	DD	AOA
Denmark 2017^(44)^	Conduct an early cost-effectiveness analysis of single-use flexible video bronchoscope technology compared to current reusable technology in a hospital intensive care setting in the United States.	8 H	EQS	AOA
Netherlands 2017^(45)^	Initially assess health technology to estimate the costs of magnetic resonance-guided high-intensity focused ultrasound ablation compared with breast-conserving treatment.	7 H and OA	MSP	AOA
United Kingdom 2017^(46)^	Establish specialist opinion and improve MED authentication technology.	10 H	MS	P
United Kingdom 2017^(47)^	Assess the Spectra Optia automated apheresis system to make recommendations.	6 H	MSP	P
Brazil 2018^(48)^	Validate the content and design of a longitudinal care program to assist adolescents in their first pregnancy.	11 H	NDD	AOA
Brazil 2018^(49)^	Validate the content of a booklet-type educational technology produced to facilitate the reception of newborns’ families in the Intensive Care Unit.	15 OA	NDD	AOA
Brazil 2018^(50)^	Analyze the quality of a virtual learning object using the Learning Object Review Instrument.	5 H	DD	STU
Sweden 2018^(51)^	Investigate the reliability with which healthcare professionals with different levels of expertise can convey the exact location of prostate cancer.	9 H	MSP	P
United States of America 2018^(52)^	Assess the cost of microprocessor-controlled knees compared to non-controlled knees.	15 H	EQS	AOA
Mexico 2018^(53)^	Define the role of metabolic surgery in 2DM treatment in Mexico.	24 H	MSP	AOA
Australia 2018^(54)^	Conduct usability testing of the app to assess pain in adults with dementia treated by paramedics; assess the suitability of the app’s algorithm as a pain assessment flowchart for use with adults with dementia; identify areas for further development in the pain app to improve user interaction; respond to recommendations made by clinical teams and work with computer science specialists to make necessary modifications.	7 H	DD	P
Mexico 2019^(55)^	Describe the design and development of a web-based system.	10 H	DD	AOA
Brazil 2019^(56)^	Validate online training for VAMOS program facilitators.	6 H	DD	P
Brazil 2019^(57)^	Validate the “*Manual de cuidados familiares com a pessoa idosa submetida à cirurgia cerebral*” educational technology.	11 H and OA	NDD	AOA
Brazil 2019^(58)^	Develop and validate the content of free web-based software (desktop and mobile applications) for self-management and personalized foot and ankle exercises for people with diabetes and diabetic neuropathy.	3 H	DD	AOA
Brazil 2019^(59)^	Design and validate the content and design of an educational game about healthy lifestyle habits for adolescents.	15 H	NDD	ADO
New Zealand 2019^(60)^	Investigate the perceived usefulness of different daily care activities for the robot, developed based on previous needs research.	19 H	DD	AOA
Sweden 2019^(61)^	Develop and assess a prototype for monitoring movements and heart rate.	NI H and OA	EQS	AOA
India 2019^(62)^	Develop a bilingual, smartphone-based educational mobile application for cardiac patients and pilot test it in an Indian clinical setting.	5 H and OA	DD	AOA
Brazil 2019^(63)^	Develop and validate the content of a free web-based software (desktop and mobile applications) for self-management and personalized foot and ankle exercises for people with diabetes and diabetic neuropathy.	19 H	DD	AOA
Brazil 2020^(64)^	Validate the “*Saúde do Homem: dicas para uma vida saudável*” educational technology content.	9 H	NDD	AOA
Brazil 2020^(65)^	Validate the suitability of the online training material and its usability to train healthcare professionals to implement the VAMOS version 3.0.	22 H and OA	DD	P
Brazil 2020^(66)^	Develop an educational intervention using a game that addresses aspects related to adolescent motherhood and childcare.	8 H and OA	NDD	ADO
United States of America 2020^(67)^	Describe the development and content of slide presentations and assess their ability to spark discussion among focus group participants.	17 H and OA	NDD	AOA
United States of America 2020^(68)^	Develop the COPE-STAR intervention using a web-based platform to help caregivers of children aged 1-5 manage their children’s symptoms and medical technology at home.	8 H	DD	AOA
Brazil 2020^(69)^	Validate the content and usability of the “*NASF em Rede*” application for teams in the Expanded Family Health and Primary Care Centers.	18 H and OA	DD	P
Brazil 2020^(70)^	Assess the technical quality of a mobile application to support nurses’ decision-making in preventing skin lesions in hospitalized newborns, according to the Product Quality Model.	20 H and OA	DD	P
United Kingdom 2020^(71)^	Develop patient-recorded indicators for manual therapy devices in epidermolysis bullosa and their use in an N-of-1 proof-of-concept study with cost analysis to investigate the performance of the disposable dressing glove compared to conventional dressings and bandages.	8 H	NDD	P
Portugal 2020^(72)^	Describe and report the initial steps in the development of a new medical device (the Duo syringe).	9 H and OA	NDD	P
Spain 2021^(73)^	Assess the maturity of the DESDE-AND classification system.	23 OA	MS	P
United Kingdom 2021^(74)^	Assess, in regional anesthesiologist specialists’ opinion, the overall performance of an Artificial Intelligence system to guide an ultrasound to confirm the correct view of the less experienced provider.	3 H	DD	AOA
India 2021^(75)^	Assess the cost-effectiveness of portable electrocardiography for cardiovascular disease screening among symptomatic and high-risk adults at a Primary Health Center.	3 H	EQS	AOA
Brazil 2021^(76)^	Develop and validate a self-care educational technology for informal caregivers.	7 H	NDD	AOA
Taiwan 2021^(77)^	Assess the cost-effectiveness of Avelumab in Taiwan.	H N	EQS	AOA
Brazil 2021^(78)^	Verify the Safe Heart mobile app content validity for monitoring and identifying heart attack risk.	10 H and OA	DD	AOA
Canada 2021^(79)^	Create a toolkit for clinicians to conduct assessments and rehabilitation on a platform enabled with immersive technologies.	NI OA	DD	AOA and P
Austria 2021^(80)^	Gain insight into the uptake, user acceptance, and concerns regarding a machine learning-based prediction application designed to improve patient safety in a clinical setting.	15 H and OA	DD	AOA
United Arab Emirates 2022^(81)^	Perform HTA for a different level of telemedicine intervention in diabetes management.	12 H	MSP	AOA
France 2022^(82)^	Highlight the organizational impacts of 3D printing in a hospital.	12 H and OA	EQS	P
Israel 2022^(83)^	Analyze the impact of two technologies that deliver MEDs directly to the brain.	8 H	DD	P
Thailand 2022^(84)^	Synthesize international documents and research on smart digital learning to improve digital health literacy; design smart digital learning; and assess its suitability.	10 H and OA	DD	AOA
Brazil 2022^(85)^	Present an initial version of the technological development and assessment of an application that supports data collection, analysis, assessment, and monitoring of healthy school cafeterias.	3 H	DD	NBC, ADO
Brazil 2022^(86)^	Produce and validate educational health video technology to encourage breastfeeding among families.	20 H	NDD	NBC
Brazil 2022^(87)^	Present the development, validation, and usability assessment of a booklet that guides training and personalizes the progression of a home-based foot and ankle exercise program.	8 H and OA	NDD	AOA
Pakistan 2022^(88)^	Develop an asynchronous virtual classroom for teaching laboratory testing factors.	17 OA	DD	STU
United States of America 2022^(89)^	Develop a virtual bariatric endoscopy simulator for training and assessment of endoscopic sleeve gastroplasty.	5 H	EQS	P
Italy 2022^(90)^	Assess the potential benefits of Magnetic Resonance-guided high-intensity focused ultrasound.	11 H	EQS	AOA
Brazil 2022^(91)^	Assess the “*Meu PICC*” ​​application usability for out-of-hospital monitoring of patients using peripherally inserted central catheters.	18 H and OA	DD	AOA
Brazil 2023^(92)^	Develop and validate an app that determines the foot risk level of patients with DM associated with health conditions.	18 H	DD	P
France 2023^(93)^	Analyze the cost-effectiveness of blinatumomab versus HC3 in children with first relapse of high-risk acute lymphoblastic leukemia.	NI H	MED	NBC
United States of America 2023^(94)^	Assess the acceptability and feasibility of a technology-driven platform to facilitate care coordination: Care4AD.	35 H	DD	AOA
Italy 2023^(95)^	Assess the cost-effectiveness of radiofrequency ablation for esophageal cancer in Italy.	5 H	EQS	AOA
Italy 2023^(96)^	Assess the benefits and added value of introducing trifluridine/tipiracil into Italian clinical practice, defining the comparison and efficacy and safety profiles in relation to other treatment options.	8 H	MED	AOA
Sweden 2023^(97)^	Assess the usability of the Electronic Health Report Form prototype to identify health and health-related problems in young people.	14 H and OA	DD	ADO
Portugal 2023^(98)^	Assess a clothing prototype that incorporates sensors for assessing pressure, temperature, and humidity to prevent pressure injuries.	9 H	NDD	P
Colombia 2023^(99)^	Determine the therapeutic classification of caplacizumab according to the Institute for Health Technology Assessment’s methodology.	5 H	MED	AOA
Italy 2023^(100)^	Assess the costs of Radiofrequency Echographic Multi Spectrometry versus conventional ionizing technology for the diagnosis of osteoporosis from the Italian National Health Service’s perspective.	6 H	MSP	AOA

*H - health; OA - other areas; MED - medication; EQS - equipment and supply; MSP - medical surgical procedure; MS - management system; NDD - non-digital device; DD - digital device; NBC - newborn/child; ADO - adolescent; AOA - adult/older adults; NI - no information; HTA - health technology assessment; DM - diabetes mellitus; P - professionals; STU - students.*

**Figure 2:**
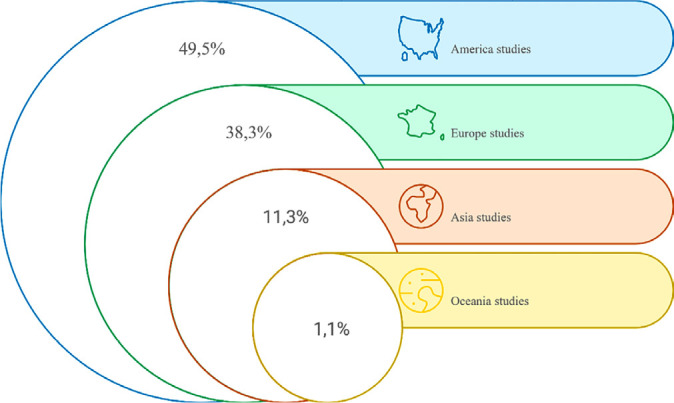
Characterization of the geographic distribution of scientific studies with the participation of specialists in health technology assessment in the 2013-2023 period

As for the countries, 44 were identified in the Americas (27 in Brazil) and 62 (69.7%) distributed in 30 countries, spanning four continents (Figure 2). In addition, 46 (51.7%) were published in the last five years (2019-2023). Regarding the typologies of technologies assessed, according to the theoretical categories^(1)^, 29 (32.6%) DDs, 19 (21.3%) MSPs, ten (11.2%) MSs, nine (10.2%) EQSs, and five (5.7%) MEDs were mapped. Furthermore, according to the empirical category that emerged, 17 (19%) non-digital devices (NDDs).

The total population of specialists across all included studies was 1,068. As for the target audiences of the technologies assessed, 52 (58.4%) were adults and older adults; 26 (29.2%) were professionals; three (3.4%) were students; three (3.4%) were adolescents; two (2.2%) were newborns and children; two (2.2%) were children and adolescents; and one (1.2%) was an adult and older adult, and professional.

**Figure 3:**
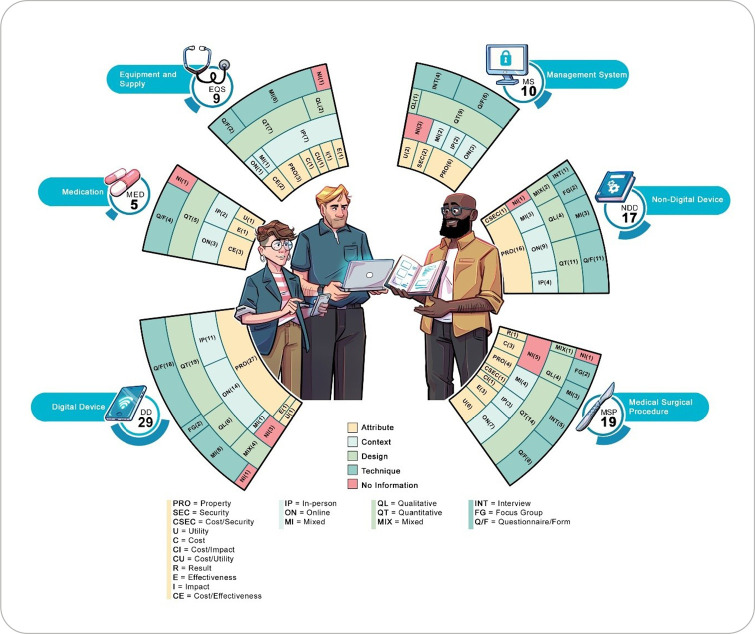
Characteristics of health technology assessment with the participation of specialists according to technology typologies

Mapping specialist participation indicated that the HTA process characteristics are related to the typologies of technologies that modify the variables analyzed, such as the attribute assessed, the context in which HTA was conducted, study design, and participation technique (Figure 3).

Concerning specialists’ areas of expertise, 57 (64%) of studies included health specialists. Only in MED assessment were all health specialists.

In EQS and MSP assessments, the majority (77.8% and 78.9%, respectively) of specialists were from the healthcare field. When specialists from other fields were included, they included members of HTA committees^(12,42,82)^. EQS assessment also included engineering participants^(61)^, and MSP assessment included participants from the cost area exclusively^(36)^ and integrated with the health area^(45)^. Regarding DDs and NDDs, the majority also included health specialists (55.2% and 64.7%, respectively). In DD assessment, there was exclusive participation of information technology specialists^(30,32,79,88)^ or information technology integrated with health specialists^(62,65,69,70,78,80,84,91,97)^. In NDD assessment, when including other areas, the design area was considered^(49,87)^ or other management^(66,67)^, pedagogy^(57)^, and information technology^(72)^ specialists were associated with health participants. In MS assessment, health specialists’ participation was not the majority (40%). When there were specialists from other areas, the area of information management^(27)^, information technology exclusively^(73)^, and information technology integrated into the health area were included^(26,28,37,40)^.

In relation to the participation of this specialist, the number of specialists per study ranged from three to 158. The sample was composed according to the type of technology assessed. It was found that the minimum number of specialists was three in EQS^(75,77)^, DD^(34,58,74,85)^, NDD^(16)^, and MSP^(42)^ assessment, and five in MED^(31)^ and MS^(27)^ assessment. The maximum numbers approached 30 in MED^(31)^, DD^(94)^, and MS^(26)^ assessment, and decreased to 20 in NDD^(86)^ assessment, and to 15 in EQS^(52)^ assessment. In MSP assessment, there was a single study^(33)^ that reached a maximum of 158.

In 11 studies, no information was found on the number of specialists^(13,15,25,29,35-38,61,79,93)^. No citations were found to justify the minimum or maximum number. Considering the sample (n=89), we found that a minimum of three and a maximum of 15 occurred in 64% of studies.

Across all typologies, the selection process took place at national and international reference institutions. The *Lattes* Platform was only mentioned as the sole source for specialist selection in studies from Brazil in NDD^(76,86,87)^ and DD^(69,70,78)^ assessment, with selection integrated into institutions in NDD^(66)^ assessment. Selection was mentioned only in the literature for EQS^(52,82)^ and MS^(21)^ assessment, integrated into institutions in MSP^(41)^ assessment. However, no information was found for all typologies (80% in MED assessment; 68% in MSP assessment; 66% in DD assessment; 65% in NDD assessment; 60% in MS assessment; 56% in EQS assessment).

Regarding the attributes assessed, the property attribute stands out, predominating in DD and NDD (93% and 88%, respectively), followed by MS (50%), EQS (33.3%), and MSP (10%). For MED, property was not assessed. Effectiveness was assessed separately in MED^(96)^, EQS^(95)^, DD^(83)^, and MSP^(23,24,100)^, and integrated with costs in MED^(19,31,93)^ and EQS^(44,75)^. Utility stood out in MSP^(13,14,35,41,47,53)^, followed by MS^(37,45)^, MED^(99)^, DD^(94)^, and NDD^(15)^. Cost assessment was performed separately MSP^(36,38,45)^ and EQS^(52)^ assessment, being integrated with utility^(77)^, safety^(72,81)^, and impact^(39)^ in EQS and MSP assessment. Safety assessment stood out in MS^(20,21)^ assessment. Impact^(82)^ and risk^(12)^ assessment were performed in EQS and MSP.

As for the context in which HTA was carried out, it was found that the online modality was the most used in NDD (53%) and DD (52%) assessment. The in-person modality was the most used in EQS assessment (77.7%). In MSP^(13,14,23,33)^, NDD^(67,71,76)^, MS^(40,73)^, EQS^(52)^, and DD^(79)^ typologies, the mixed modality stood out, in which both the online and in-person modalities were used.

The designs of HTA studies in which specialists participated were mostly quantitative in all typologies: MED (100%); MS (90%); EQS (77.7%); MSP (73.7%); DD (65.5%); and NDD (64.7%). Qualitative designs were not identified only in MED assessment, and mixed methods studies were used in MSP^(24)^, NDD^(72,98)^, and DD^(80,85,88,97)^ assessment.

The most commonly used techniques for specialist participation were structured instruments (questionnaires and forms), reaching 48% in DD assessment studies, 47% in NDD, and 36% in MSP. In DD assessment, the diversity of techniques combines the questionnaire with thinking aloud^(29,30)^, use of an application^(85)^, focus group alone^(43,80)^ and combined with testing and Delphi panel^(53)^, exposure followed by handling and assessment instrument^(32)^, in addition to interview after technology presentation^(60)^, prototype and scoring handling^(62)^, software use, and tests based on case studies^(70)^ and simulation^(79)^.

In MS^(20,21,28,37)^ and DD^(16)^ assessment, the use of interviews alone stood out. In EQS assessment, interviews combined with focus groups^(82)^, questionnaires^(90)^, and telephone discussion panels^(52)^ were used. Also, in EQS assessment studies, other combined techniques were identified, such as regular meetings, focus groups, workshops^(61)^, use of models and questionnaires^(77)^, procedural filming, and questionnaires^(89)^.

In MED assessment, the use of modified Delphi stood out^(31,99)^. In NDD and MSP assessment, the use of isolated techniques such as focus groups^(12,17,72,81)^, modified Delphi^(33)^ and interviews^(23,24,38,53,100)^ was also verified. In NDD assessment, combined techniques were used, such as meetings and collaborative design, with in-person discussions and telephone meetings^(67)^, meetings, email exchanges^(71)^, questionnaires, and focus groups^(98)^. In MSP assessment, case study analysis and focus groups^(13)^, interviews, questionnaires^(14)^, plotting and drawings were used^(51)^.

## DISCUSSION

The available evidence mapped in this scoping review showed that the participation of specialists in HTA processes varies according to the types of health technologies assessed. These characteristics vary for each type assessed regarding the area of expertise, assessed attribute, selection, sample, context, design, and technique.

One characteristic of HTA is that a variety of specialties participate, and there are different conceptions of who is considered a specialist. Content specialists with knowledge of technology stand out in assessment processes, and they may come from different areas of specialization, but primarily from the healthcare field, as do method specialists who will contribute to quality assurance^(8)^. The mapping indicated the participation of specialists from fields other than healthcare, such as engineering, IT, design, pedagogy, and others. A recommendation for future studies is to map challenges by field, including nursing.

Another characteristic is the variety of strategies. The studies assessed different types of devices, including digital, non-digital, and procedural, which is consistent with recommendations from Chinese researchers, who state that devices differ considerably from pharmaceuticals in many ways, making HTA challenging. The authors call on both academic communities and agencies to standardize the process, methodologies, and criteria for HTA on devices, with a view to expanding assessments, as current HTA guidelines still focus primarily on MEDs^(101)^.

Despite the challenges of HTA processes, the variety of specialties and strategies favors the collection of diverse analyses and contributions, which points to the triangulation of participant areas, designs, and techniques. Triangulation is a strategy for improving studies by involving different perspectives and used to increase their credibility by involving two or more aspects and enabling the understanding of the evaluative phenomenon at different levels, considering the complexity of the objects of study (health technologies) and the conditions of the assessment process itself^(102)^.

In this process, regarding specialists’ areas of expertise, it became clear that there are studies involving only healthcare specialists, studies in which healthcare participants are integrated into different areas, and studies in which other areas participate in isolation. It was also found that, in all technology typologies assessed, healthcare specialists participated, particularly in the MED typology, where healthcare was the only specialty in all studies.

Healthcare specialists participated, either individually or in conjunction with other fields, primarily in the HTA of technologies, with the exception of MS. The specialist’s field of expertise depends on the type of technology being assessed. Among the European Union, the United Kingdom, Canada, and Australia, healthcare and other field specialists participate in HTA studies. The participation of specialists from different fields can ensure transparency and the inclusion of reflections from diverse perspectives^(103)^.

In a study where specialists provided estimates for technical issues, both in their area of expertise and in areas where they were not, it was found that participants were more serious and cautious about tasks in studies directly related to their specialties. Therefore, the specialists’ area of expertise influences HTA. The recommendation is that, for any HTA process, all specialists have the necessary substantive experience and relevant knowledge of the topic under assessment^(104)^.

Regarding the sample in HTA studies, it was possible to map the number of specialists, as well as verify the minimum and maximum number, which indicated a variation between three and 15. Furthermore, no mention was found in the studies of standards or models that determine the number of specialists to be reached, which reinforces the assertion that there is no established standard regarding the number of specialists^(105)^.

A study that considered 22 HTA organizations and included 42 documents on the specialist sample found that participation, whether individual or group, varied in number of members. Some agencies recommend including more than one specialist to obtain a range of views. It is noteworthy that the size of the specialist panels indicated in the Irish guideline ranges from five to 20 individuals^(8)^, which converges with the mapping carried out in this review.

When mapping evidence on specialist selection, a high number of studies found no information on the sources consulted for specialist selection, with the highest percentage being in MED assessment studies (80%). It is understood that selection is a challenging task for assessment studies, and researchers must establish both quantitative and qualitative criteria to achieve reliable results. Regarding specialist selection, it is important to emphasize that accurate analysis of a technology’s characteristics is related to specialists’ confidence and experience in the field, and they must be able to analyze, interpret, and infer quantitative and/or qualitative values^(105)^.

Regarding the mapping of evidence regarding the attribute assessed by specialists, studies were identified in which properties were assessed (content, appearance, usability, quality), which characterizes an assessment in the first life cycle of the technology, before registration, when technical and operational aspects of the technology are verified. In these studies, when the property assessed was content and appearance, the term “validation” was mostly used, while in studies in which the property assessed was usability and quality, the term was “assessment”.

Regarding the attributes focused on outcome aspects, more characteristic of the second life cycle onwards, studies assessing effectiveness, utility, costs, safety, impact, and risks were found. It is considered relevant that in HTA, studies are carried out at different points in the life cycle of a health technology^(106)^, as the process is characterized by being systematic and contemplating both properties and effects and/or impacts, with a view to improving the quality and value of the technology, thus facilitating decisions^(103)^.

Historically, HTA has focused on the safety and efficacy of an intervention, as well as its cost-effectiveness, although there is a growing awareness that a broader form of HTA is needed. It is noteworthy that some efforts are already underway, albeit limited to a few countries, to address this need. If HTA fails to expand its assessment and recommendations, without including other issues such as properties, for instance, it may be considered irrelevant to the of decision-makers’ real needs. HTA should (and increasingly does) have recommendations on technologies that go beyond safety, efficacy, and cost-effectiveness^(107)^, which was evident in the monitoring carried out. Safety, one of the attributes identified in the mapping, particularly in MS assessment studies, where it was assessed both separately^(20)^ and integrated with risks^(21)^, seeks to ensure that health technologies are analyzed to minimize risks to patients and optimize desired outcomes^(108)^.

Regarding context, which refers to the environments used for specialists to interact with research teams (online or in-person), evidence converged toward a multidimensional perspective. In the NDD and DD typologies, online was predominant, and in the EQS typology, in-person was predominant. Further evidence suggests that both contexts were used in studies assessing the MSP^(13,14,23,33)^, NDD^(68,71,76)^, MS^(40,73)^, EQS^(52)^, and DD^(79)^ typologies.

We understand that both contexts have positive and negative aspects. Authors acknowledge that conducting a in-person specialist consultation requires significant effort, access to resources, training, and time, especially when multiple specialists are involved, as in HTA studies. It can be very time-consuming, especially in disease areas where there are few specialists and many are geographically dispersed and have busy schedules. Furthermore, during an in-person consultation, the facilitator must be careful not to impose their beliefs or biases on the specialist. By using the online environment, more specialists can be reached in a shorter time, but generally, the cost of very low response rates, especially when targeting physicians, tends to reduce data collection costs and the level of recording errors^(104,109)^.

In relation to the study designs in which specialists participated, only the MED assessment did not use qualitative assessment. In the remaining studies, in addition to quantitative and qualitative studies, mixed methods studies were also identified in MSP^(24)^, NDD^(72,98)^, and DD^(80,85,88,97)^ assessment.

Specialist opinion can be qualitative, used to validate simulated health pathways in a cost-effectiveness model, or quantitative, used to provide estimates, for instance. The more structured consultation aims to facilitate the process of obtaining quantitative data in order to obtain opinions that are as rigorous and scientific as possible. These methods allow specialists to specify a quantity of interest, as well as the associated uncertainty surrounding it, which can be encoded as a probability distribution^(110)^. The predominantly quantitative HTA design across all technology typologies is consistent with the attribute being assessed. For instance, in the MED typology, the primarily assessed attribute was cost-effectiveness, which requires a quantitative design. Another example is the quantitative designs in property assessments, which primarily mapped the use of Content Validity Index calculations.

Regarding the techniques applied to specialists, the diversity of modalities stood out, both individually and in combination. In all typologies, questionnaires and forms were used, as well as focus groups, interviews, and the Delphi method, which is recommended for gathering specialist opinions^(8)^. Specifically regarding the Delphi method, there are numerous characteristics that are considered advantageous, such as: anonymity among participants, which avoids embarrassment, inhibitions, and intimidation that can occur in in-person meetings, allowing everyone to express their comments, even those specialists whose opinions are minority; the possibility of providing participants with feedback on their contributions; the possibility of reviewing specialists’ responses, as well as the formation of heterogeneous groups, when the subject or product to be assessed is multidimensional and multidisciplinary. When applied in a virtual environment, there is another difference and advantage to its use, as it allows the participant more time to reflect on the technology assessed, which can lead to greater adherence, participation, in addition to lower costs and time spent on its implementation^(105,111)^.

We believe it is important to adopt different techniques in HTA studies, as they allow for the collection of both objective and subjective perspectives. Opinions gathered through individual interviews (conducted in person or remotely) or through written interviews with answers to specific questions facilitate the analysis of different perspectives expressed by specialists^(110)^. In relation to questionnaires, in relation to the context in which they are applied, it is worth noting that, in the online environment, they present a greater return than those self-completed in person, allowing them to reach a wider geographic area; another advantage is that there is no pressure for the researcher to be present, as anonymity can be fully preserved, and there is a lower cost than carrying out, for instance, an interview or focus group^(109,112,113)^.

Focus groups encourage the exchange of experiences, insights, and impressions, which can enable collective reflection on the technology being assessed. The technique creates an environment that allows for problematization and in-depth discussions, enabling, above all, knowledge and understanding of subjects’ distinct expertise. The questions raised about the technology in question can spark reflection on the assessment process itself and elicit new meanings for the assessment experience itself^(114,115)^.

This result highlighted a gap in HTA studies regarding the lack and/or incompleteness of information that characterizes specialists’ participation in the HTA process, especially regarding selection, context and technique used.

### Study limitations

The limitation of this review was the non-development of the third stage of evidence search and selection, which refers to the manual search in the list of references of studies included in the sample.

### Contributions to nursing

The review contributes to the healthcare field by offering a comprehensive analysis of specialist participation in the assessment of various health technologies. These findings emphasize the need for a more structured approach to assessment based on the specific characteristics of each technology typology, providing insights for implementing innovations in this sector. Thus, the review not only expands knowledge about specialist participation but also guides future research and practice in healthcare.

## CONCLUSIONS

Mapping the evidence on specialist participation in the HTA process showed that the attribute assessed is one of the defining characteristics of specialist participation and is directly related to the type of technology. The predominant area of expertise for inclusion in assessment is healthcare, with the exception of technologies classified as MS, and the inclusion of other areas depends on the type of technology being assessed. The sample of specialists ranged, in most studies, from three to 15. Specialists were selected based on information obtained from institutions and the literature, but this information is lacking, as there is little transparency in studies of this data.

## Data Availability

The research data are available within the article.
