# Supplementary material for: Expert participation in the health technology assessment process: a scoping review
Source: Rev Bras Enferm. 2026 Mar 16;79:e20250157. doi: 10.1590/0034-7167-2025-0157 (PMC12995334; doi:10.1590/0034-7167-2025-0157)
Supplement: Translation

# Translation: Participação de especialistas no processo de avaliação de tecnologias em saúde: revisão de escopo

**DOI:** 10.1590/0034-7167-2025-0157pt

**Authors:** Sandra Maria dos Santos Figueiredo, Fernanda de Nazaré Almeida Costa, Marcia Helena Machado Nascimento, Cristiane Cardoso de Paula, Liane Bahú Machado, Eliana Rosa da Fonseca, Elizabeth Teixeira

**Affiliations:** I Centro Universitário do Estado do Pará. Belém, Pará, Brasil; II Universidade do Estado do Pará. Belém, Pará, Brasil; III Universidade Federal de Santa Maria. Santa Maria, Rio Grande do Sul, Brasil; IV Universidade Federal do Rio de Janeiro. Rio de Janeiro, Rio de Janeiro, Brasil

**Keywords:** Avaliação da Tecnologia Biomédica, Revisão por Pares, Tecnologia Biomédica, Equipamentos e Provisões, Métodos.

## Abstract

**Objetivos:**

mapear evidências sobre a participação de especialistas no processo de avaliação de tecnologias em saúde.

**Métodos:**

revisão de escopo pelo método JBI, com busca nas bases de dados MEDLINE/PubMed, Scopus, EMBASE/Elsevier, WoS/Clarivate, CINAHL, ASP/EBSCO, APA PsycINFO, ERIC, LILACS/BVS, SciELO e INAHTA. Foram considerados artigos originais publicados de 2013-2023. Dados foram extraídos de 89 estudos, analisados com estatística descritiva.

**Resultados:**

entre três e 15 especialistas (64% dos estudos) participaram. A avaliação do atributo propriedade ocorreu em dispositivos digitais, não digitais, sistemas de gestão, equipamentos (93,1%, 94,2%, 60%, 33,3%, respectivamente), do atributo custo-efetividade, em medicamentos (60%), e do atributo utilidade, em procedimentos médico-cirúrgicos (31,6%). A participação foi *online* em dispositivos digitais e não digitais (53% e 52%, respectivamente), presencial, em equipamentos (77,7%), e mista, nas demais tipologias.

**Conclusões:**

é necessário estruturar a avaliação de acordo com as características específicas da tipologia de tecnologia, fornecendo *insights* para implementação de inovações nesse setor.

## INTRODUÇÃO

As tecnologias em saúde são classificadas segundo natureza do material: medicamento (MED); equipamento e suprimento (EQS); procedimento médico-cirúrgico (PMC); sistema de gestão (SG); e dispositivo digital (DD)^(1)^. MED e EQS são consideradas como tecnologias biomédicas, que são aquelas que interagem diretamente com os usuários; PMC constitui as tecnologias médicas, que são essenciais para qualidade na aplicação das tecnologias biomédicas, como técnicas cirúrgicas, normas técnicas, entre outras; SG se refere a sistemas de suporte organizacional, que englobam uma estrutura de apoio técnico e administrativo, e sistemas de informação e organização da prestação da atenção à saúde, que, junto com as tecnologias médicas, compõem as tecnologias de atenção à saúde; e DD é intervenção acessível por meio de dispositivos móveis. Existem outras classificações quanto ao propósito da tecnologia e estágio de difusão^(1)^, inclusive tipologias utilizadas pela enfermagem brasileira.

Os ciclos de vida de uma tecnologia são inovação, difusão inicial, incorporação, utilização em larga escala e abandono. O processo de inovação tecnológica (primeira fase do ciclo) começa com a invenção de um novo produto, processo, ou prática, e se encerra por ocasião da primeira utilização prática (difusão inicial - segunda fase do ciclo)^(2)^. A avaliação se dá tanto na fase de desenvolvimento ou criação do produto (anterior ao registro) quanto na fase de incorporação e utilização do produto (após a aplicação).

Entretanto, o processo de avaliação evolui para abordagens mais construtivas desde o início da tecnologia até sua obsolescência. Portanto, novos conceitos estão sendo aplicados, como aconselhamento científico, diálogo precoce, observação pós-introdução de tecnologias em saúde adequação, reavaliação e desinvestimento, entre outros^(2)^. Está em discussão a adoção do conceito de “abordagem de ciclo de vida” mais completa e holística, que pode promover o engajamento das partes interessadas e a geração de evidências robustas^(3)^.

A avaliação de tecnologias em saúde (ATS) é um processo multidisciplinar que utiliza métodos explícitos para determinar o valor de uma tecnologia em saúde em diferentes pontos de seu ciclo de vida. O objetivo é informar a tomada de decisão para promover um sistema de saúde equitativo, eficiente e de alta qualidade^(4)^.

A maioria das ATSs se concentra na medicina clínica e em produtos farmacêuticos, resultando em uma escassez de avaliações voltadas para intervenções em saúde pública^(5)^. A avaliação de intervenções complexas na saúde pública enfrenta desafios, incluindo a heterogeneidade das evidências, que decorre da diversidade metodológica dos estudos. A ATS contribui para essa legitimidade com conteúdo e procedimentos, com o conteúdo sendo a evidência robusta e confiável que será usada para tomada de decisão informada, enquanto os procedimentos se relacionam aos princípios de boa governança que, em última análise, constroem confiança^(6)^.

Destaca-se que, em todas as fases e ciclos de vida de uma tecnologia, diferentes atores podem participar no processo de avaliação. Cada etapa do processo de ATS utiliza diferentes fontes de informação e envolve diferentes grupos de pessoas^(7)^. O especialista no processo de ATS se refere aos indivíduos externos, não sendo integrantes habituais da equipe de ATS, que detêm conhecimento ou *expertise* especializada em um domínio pertinente. O termo “opinião de especialista” abrange as contribuições que esses indivíduos podem oferecer ao processo de ATS, incluindo dados quantitativos e qualitativos, bem como experiências e avaliações de valor^(8)^.

Para verificar a necessidade e exequibilidade do mapeamento de experiências metodológicas e lacunas neste campo da participação de especialistas no processo de ATS, foi desenvolvida uma busca preliminar de protocolos e/ou relatórios de revisões. Considerando as fontes de informação *Medical Literature Analysis and Retrieval System Online* (MEDLINE)/PubMed, *Cochrane Database of Systematic Reviews, JBI Evidence Synthesis, International Prospective Register of Systematic Reviews* e *Open Science Framework* (OSF), foram identificadas 2.197 referências. Essa pré-análise identificou uma revisão de diretriz que reforça a importância de se considerar o uso da opinião de especialistas na ATS, com menção que, apesar das recomendações existentes, há escassez de relatos sobre os métodos de elicitação de especialista^(8)^. A participação de especialistas ocorre por meio de diversas modalidades, as quais podem ser sistematicamente mapeadas na literatura, favorecendo a tomada de decisão metodológica em estudos envolvendo especialistas na ATS.

## OBJETIVOS

Mapear as evidências sobre a participação de especialistas no processo de ATS.

## MÉTODOS

### Aspectos éticos

Por se tratar de estudo secundário, com extração de dados disponíveis na literatura, não houve a necessidade de apreciação ética. Destaca-se que foram respeitados os direitos autorais com devida citação e referenciamento dos estudos primários.

### Desenho do estudo

Trata-se de revisão de escopo, conduzida a partir do método do JBI^(9)^. Para qualidade e transparência da redação, foi aplicado o *Preferred Reporting Items for Systematic reviews and Meta- Analyses extension for Scoping Reviews* ^(10)^. O protocolo foi registrado no OSF (https://doi.org/10.17605/OSF.IO/RE8TW).

### Estratégia de busca e fonte de dados

Adotou-se o mnemônico PCC para estruturar a questão da revisão e o mapeamento de termos, para composição da estratégia de busca nas fontes de informação: População (P): especialistas; Conceito (C): ATS; Contexto (C): qualquer cenário da área da saúde. A pergunta de revisão foi: quais as características da participação de especialistas em processo de ATS?

A estratégia de busca foi estruturada por uma bibliotecária com *expertise* em estudos de revisão na área da saúde. Para elaboração da estratégia, foi desenvolvido o mapeamento de termos utilizando, primeiramente, os descritores e palavras utilizadas na busca preliminar, o qual foi ampliado com a pré-análise dos documentos identificados, incluindo outros termos contidos nos títulos, resumos e termos de indexação. Assim, foi estruturada a estratégia de busca aplicada ao MEDLINE/PubMed: (Specialist*[tiab] OR expert*[tiab] OR “Consensus”[mh] OR Consensus[tiab] OR “Consensus Development”[tiab] OR “expert committee”[tiab] OR “expert panel”[tiab] OR “expert elicitation”[tiab] OR “effectiveness assessment”[tiab] OR “expert views”[tiab] OR “expert committee”[tiab] OR “expert panel”[tiab] OR “expert elicitation”[tiab] OR “Delphi Technique”[mh] OR “Delphi Method”[tiab] OR “Delphi Studies”[tiab] OR “Delphi Study”[tiab] OR Delphi Technic*[tiab] OR Delphi Technique*[tiab])) AND (“Technology Assessment, Biomedical”[mh] OR “Biomedical Technology Assessment”[tiab] OR (“Biomedical Technology”[mh] AND assessment*[tiab]) OR Health Technology Assessment*[tiab] OR HTA[tiab] OR ((Health Technolog*[tiab] OR Health Care Technolog*[tiab] OR Health Technolog*[tiab] OR Medical Technolog*[tiab] OR care technolog*[tiab] OR caring technolog*[tiab]) AND (assessment*[tiab])))) AND (((2013:2022[pdat]) AND (English[Filter] OR Portuguese[Filter] OR Spanish[Filter]))). Esta estratégia foi adaptada para cada fonte de informação.

A busca bibliográfica foi realizada em 19 de junho de 2023 nas seguintes fontes de informação: MEDLINE (PubMed); Scopus (Elsevier); *Excerpta Medica dataBASE* (EMBASE (Elsevier)); Literatura Latino-Americana e do Caribe em Ciências da Saúde; *Índice Bibliográfico Español en Ciencias de la Salud*; Base de Dados de Enfermagem; *Western Pacific Region Index Medicus; MULTIMEDIA; Centro Nacional de Información de Ciencias Médicas de Cuba; World Health Organization Library & Information Networks for Knowledge; Literatura Peruana en Ciencias de la Salud*; IRIS PAHO; Base Internacional de Guias GRADE; *PREPRINT*-medRxiv; ARGMSAL; *Bibliografía Nacional en Ciencias de la Salud Argentina*; Localizador de Informação em Saúde; Políticas Informadas por Evidências; ColecionaSUS; Secretaria de Estado da Saúde de São Paulo; Campusvirtualsp_brasil; Bibliografia Brasileira de Odontologia; IndexPsi; *African Index Medicus*; DESASTRES; *PREPRINT*-*Scientific Electronic Library Online* (SciELO) (Biblioteca Virtual em Saúde (BVS)); *Education Resources Information Center* (ERIC) *Core Collection* (*Clarivate Analytics*); *Cumulative Index to Nursing and Allied Health Literature and Academic Search Premier* (EBSCO); *American Psychological Association* (APA PsycINFO); *International HTA Database*; e SciELO.

Após a busca, foram verificadas as duplicatas no gerenciador de referências EndNote 20/2020 (*Clarivate Analytics*, PA, EUA).

### Critérios de elegibilidade

Os estudos incorporados foram analisados para inclusão ou exclusão partir do PCC, sendo assim definidos: População (P) - Incluídos estudos em que tiveram participação de especialistas em alguma etapa do processo de ATS: pessoas externas à equipe de pesquisa com conhecimentos ou experiência em uma área relevante e que tenham tido participação individualmente, em painel ou em comissão, nos diferentes momentos do processo, ou seja, em alguma das etapas de ATS. Conceito (C) - Incluídos estudos de ATS em que se determinou o valor de uma tecnologia de saúde em diferentes pontos do seu ciclo de vida segundo distintos métodos, desenhos, técnicas e instrumentos, em que foram considerados aspectos clínicos, sociais, éticos e econômicos, podendo incluir evidências de propriedades internas, segurança, eficácia, resultados relatados pelo paciente, eficácia (no mundo real), custo e custo-efetividade, bem como impactos sociais, legais, éticos e políticos. Contexto (C) - Incluídos estudos realizados em qualquer cenário da área da saúde, sem delimitar o local onde foi desenvolvida a ATS, a fim de não limitar a busca e seleção e possibilitar o mapeamento dessa característica nas produções.

Foram incluídos artigos originais, nos idiomas inglês, português ou espanhol, publicados a partir de 2013, considerando o marco da Organização Pan-Americana da Saúde^(11)^ de estimular o estabelecimento de processos decisórios para incorporação de tecnologias em saúde baseadas na ATS. Foram excluídos estudos de revisão, editoriais, cartas ao editor, estudos de tradução e adaptação cultural de instrumentos e estudos em que a participação se deu para avaliar estratégias ou parâmetros ou diretrizes para ATS.

### Seleção de estudos, extração dos dados, análise e apresentação dos resultados

Como critério de qualidade, para minimizar a chance de erro, as etapas de seleção e de extração foram desenvolvidas de modo duplo independente, e quando não houve consenso, um terceiro revisor foi consultado para tomada de decisão. Os revisores foram treinados quanto aos critérios de elegibilidade. Houve uma pilotagem tanto do *checklist* de seleção (critérios de inclusão e de exclusão) quanto do instrumento de extração.

A etapa de seleção foi desenvolvida na plataforma Rayyan^®^ (http://rayyan.qcri.org), desenvolvida pelo *Qatar Computing Research Institute*. A primeira etapa de seleção foi por leitura de títulos e resumos entre outubro de 2023 e janeiro de 2024. Os artigos que atenderam aos critérios de elegibilidade passaram para a segunda etapa, e foram lidos na íntegra para a segunda fase de seleção, o que se deu entre fevereiro e abril de 2024. Na plataforma Rayyan^®^, foram utilizados aplicativos *Blind ON* ou *Blind OFF*, que permitiram gerenciar o cegamento e a verificação de conflitos.

A extração de dados foi desenvolvida, conforme o instrumento do JBI, em uma planilha do *Microsoft Excel* ^®^ estruturada com duas abas, coerente com o método do JBI, que se refere à caracterização (aba 1: ano; país; objetivo; público-alvo da tecnologia) e resposta ao escopo de mapeamento (aba 2: características da ATS, como tipologia da tecnologia, atributo avaliado, contexto delineamento e técnica de coleta de dados, e características dos especialistas, como área, amostra e seleção).

A tipologia partiu inicialmente de uma classificação teórica conforme a Organização Mundial da Saúde (OMS)^(1)^: MED; EQS; PMC; SG; e DD. A tipologia DND emergiu do mapeamento e foi incluída posteriormente, caracterizando-se como classificação empírica.

Na análise, os dados foram verificados segundo estatística descritiva para apresentação dos resultados da caracterização dos estudos: tendência temporal; distribuição geográfica; e população-alvo. A apresentação dos resultados foi tabulada e acompanhada de resumo narrativo, além de gráficos temporais e geográficos, e para comparação, foram elaborados no *Napkin* e *Photoshop*.

## RESULTADOS

Foram identificados os seguintes quantitativos de referências, perfazendo um total de 8.938 estudos: 1.172 na BVS; 733 na EBSCO; sete na ERIC; 1.094 na EMBASE; 570 na *HTA Database*; 2.015 na APA PsycINFO; 1.056 na *Web of Science*; 1.869 na Scopus; 100 na SciELO; 2.015 na PubMed.

Desses, 4.456 foram excluídos por duplicidade, e 4.482 foram selecionados para leitura de título e resumo. Na sequência, 4.122 foram excluídos por não contribuírem com a questão de pesquisa. Assim, 360 foram selecionados e, 29, eliminados, por não estarem acessíveis na íntegra. Foram lidos 331, sendo excluídos 242. Por fim, foram incluídos nesta revisão 89 estudos (Figura 1).

**Figura 1:** Fluxograma do *Preferred Reporting Items for Systematic reviews and Meta- Analyses extension for Scoping Reviews* 2020 para identificação e inclusão de estudos

Foi desenvolvido o mapeamento das características das produções sobre a participação de especialistas no processo de ATS referente à localização geográfica, temporal, amostra, área de conhecimento, tipologia da tecnologia avaliada e público-alvo da tecnologia (Quadro 1).

**Quadro 1:** Caracterização dos estudos primários incluídos na revisão em ordem cronológica de publicação, Belém, Pará, Brasil, 2024

País/ano	Objetivo	Amostra/área	Tipo	Público
Irã 2013^(12)^	Comparar fatores do paciente, segurança e consideração organizacional para avaliação de tecnologia em saúde da nova biotecnologia *vital pulp therapy*/*calcium enriched mixture* comparada com *root canal therapy*.	6 S e OA	PMC	AI
Reino Unido 2013^(13)^	Avaliar tratamento para úlceras venosas nas pernas associado a bandagens e roupas de compressão.	SI S	PMC	AI
Reino Unido 2013^(14)^	Investigar semelhanças e diferenças entre *Hydration Response Technology* e terapia de pressão negativa para feridas com relação à preparação do leito da ferida e elaborar recomendações para uso.	23 S	PMC	AI
Itália 2013^(15)^	Avaliar o estado tecnológico geral dos clipes mitrais no processo de tratamento da regurgitação mitral, com particular referência aos métodos tradicionais, além de contextualização das análises dentro da estrutura hospitalar, identificando questões de criticidade e melhorias.	SI S	DND	AI
Croácia 2013^(16)^	Desenvolver, implementar e avaliar cartas terapêuticas de pacientes escritas por alunos como uma ferramenta no ensino de medicina de família.	3 S	DND	EST
Espanha 2013^(17)^	Concordar entre os especialistas sobre o conteúdo de nova educação materna baseada nas necessidades de saúde da mulher, e analisar os modelos teóricos e estratégias educativas maternas para responder a essas necessidades.	11 S	DND	P
Brasil 2013^(18)^	Descrever o processo de avaliação de tecnologia educativa de literatura de cordel com relação aos aspectos de conteúdo sobre amamentação.	6 S	DND	AI
Argentina 2013^(19)^	Avaliar a relação custo-efetividade da *Oxytocin in Uniject* na América Latina e Caribe.	8 S e OA	MED	AI
Cuba 2014^(20)^	Abordar uma ferramenta informática que ajuda na determinação de ações de saúde relacionadas à hipertensão arterial na Atenção Primária à Saúde através do processamento analítico em linha e da razão baseada em casos.	9 S	SG	AI
Alemanha 2014^(21)^	Discutir as perspectivas, demandas e preocupações de segurança e risco associadas aos *lab-on-a-chip systems*.	30 S	SG	AI
Brasil 2014^(22)^	Elaborar e obter consenso sobre tecnologia assistencial para avaliação clínica diária de pacientes no pós-operatório, partindo de suporte teórico e adequando à dinâmica dos hospitais.	9 S	PMC	AI
Reino Unido 2014^(23)^	Determinar a estratégia de teste diagnóstico mais econômica para o diagnóstico e tratamento de heavy menstrual bleeding e post-menopausal bleeding	14 S	PMC	AI
Reino Unido 2014^(24)^	Identificar e descrever a intervenção *selective decontamination of the digestive tract*, os pontos de vista sobre a base de evidências, a aceitabilidade de pesquisas futuras, e a viabilidade de novos ensaios clínicos randomizados.	36 S	PMC	AI
Peru 2015^(25)^	Produzir um serviço de tecnologia da informação *online* para a saúde e ajudar a tornar os processos de avaliação e aquisição de equipamentos médicos informados de acordo com mercado e necessidades das instituições de saúde.	SI S	SG	P
Brasil 2015^(26)^	Avaliar desempenho funcional e qualidade técnica do Sistema de Documentação Eletrônica do Processo de Enfermagem.	37 S e OA	SG	P
Reino Unido 2015^(27)^	Explorar e promover melhorias na produção de evidências para a Avaliação de Tecnologias em Saúde, na área de medicamentos considerando a necessidade dos diferentes stakeholders ao longo de todo o ciclo de vida das tecnologias em saúde	75 S	MED	P
Espanha 2015^(28)^	Identificar as necessidades do usuário de uma nova tecnologia médica para gerenciar e monitorar pacientes com doença de Parkinson e usar essas necessidades do usuário para uma avaliação preliminar de um sistema específico chamado PERFORM com estudo de caso.	16 S e OA	SG	AI
Brasil 2015^(29)^	Apresentar o aplicativo *Diabetes Food Control*, para avaliar os marcadores do consumo alimentar dos diabéticos, baseado em questionário validado.	SI S	DD	AI
Brasil 2015^(30)^	Apresentar o *Alz Memory*, um jogo desenvolvido para estimular a memória de pacientes com Alzheimer, com vistas a minimizar os efeitos da doença.	10 OA	DD	AI
Argentina 2015^(31)^	Avaliar a relação custo-efetividade da ocitocina na América Latina e Caribe.	30 S	MED	AI
Brasil 2016^(32)^	Avaliar a usabilidade de protótipo educacional digital sobre um novo método para monitoração da pressão intracraniana de forma minimamente invasivo para enfermeiros e médicos.	4 OA	DD	P
Espanha 2016^(33)^	Conhecer os critérios dos especialistas em hipertensão sobre combinações triplas em doses fixas e explorar possíveis diferenças de critérios quanto à sua utilização entre médicos de três especialidades envolvidos no controle da pressão arterial: medicina interna, cardiologia e nefrologia.	158 S	PMC	AI
Brasil 2016^(34)^	Desenvolver e avaliar preliminarmente um aplicativo para dispositivos móveis que auxiliem profissionais da voz no gerenciamento da saúde vocal.	3 S	DD	P
Estados Unidos da América 2016^(35)^	Determinar se a aplicação do método *Headroom* combinado com a análise de retorno sobre o investimento permite estimar a viabilidade comercial potencial de dispositivo terapêutico e cinco dispositivos de diagnóstico.	SI S	PMC	P
Holanda 2016^(36)^	Avaliar a eficácia de custo a curto prazo de quatro testes diagnósticos complementares em pacientes com artrite inflamatória precoce em risco de artrite reumatoide.	SI OA	PMC	AI
Índia 2016^(37)^	Relatar o processo de implementação de *Mother and Child Tracking System* para rastreamento materno-infantil baseado em *tablet* em Bihar.	SI S e OA	SG	P
Finlândia 2016^(38)^	Analisar a fase de adoção de tecnologia em saúde.	SI S	PMC	P
Polônia 2016^(39)^	Avaliar os custos e as consequências do uso de uma tecnologia médica inovadora, e o inserto vaginal de misoprostol, para indução do parto, no lugar de tecnologias alternativas usadas como padrão de tratamento.	5 S	PMC	AI
Colômbia 2017^(40)^	Validar o impacto da ferramenta de auditoria para gerenciamento de tecnologia biomédica.	12 S e OA	SG	P
Suécia 2017^(41)^	Quão preciso é o diagnóstico remoto baseado em imagens de queimaduras comumente apresentadas em centros de emergência de *Western Cape*, quando visualizado em um dispositivo portátil? As avaliações remotas têm precisão comparável quando feitas em dispositivos portáteis em comparação com uma tela de computador?	26 S	PMC	P
Espanha 2017^(42)^	Descrever a variedade de acordos, métodos e processos de ATS na adoção e uso de uma intervenção preventiva controversa em uma seleção de países das regiões Europa Central, Oriental e Sudeste, e América Latina e Caribe, além de implementação de vacinas em países de renda média-baixa e fornecer recomendações àqueles países.	3 S e OA	PMC	RNC e ADO
China 2017^(43)^	Desenvolver uma nova aplicação móvel para informação e apoio social e emocional às mulheres com câncer da mama em quimioterapia para promover a autoeficácia e apoio social, melhorando as estratégias de gestão dos sintomas.	8 S	DD	AI
Dinamarca 2017^(44)^	Realizar análise precoce de custo-efetividade da tecnologia de videobroncoscópio flexível de uso único em comparação com a atual tecnologia reutilizável em um ambiente de terapia intensiva hospitalar nos Estados Unidos da América.	8 S	EQS	AI
Holanda 2017^(45)^	Avaliar inicialmente tecnologia de saúde para estimar os custos da ablação por *magnetic resonance-guided high intensity focused ultrasound*, em comparação com o tratamento conservador da mama.	7 S e OA	PMC	AI
Reino Unido 2017^(46)^	Estabelecer a opinião de especialistas e melhorias para a tecnologia de autenticação de MEDs.	10 S	SG	P
Reino Unido 2017^(47)^	Avaliar o sistema automatizado de aférese *Spectra Optia* para fazer recomendações.	6 S	PMC	P
Brasil 2018^(48)^	Validar o conteúdo e a aparência de um programa de cuidado longitudinal para assistir adolescentes em sua primeira gravidez.	11 S	DND	AI
Brasil 2018^(49)^	Validar o conteúdo de tecnologia educacional do tipo cartilha, produzida para mediar o acolhimento de familiares de recém-nascidos na Unidade de Terapia Intensiva.	15 OA	DND	AI
Brasil 2018^(50)^	Analisar a qualidade de um objeto virtual de aprendizagem a partir do *Learning Object Review Instrument*.	5 S	DD	EST
Suécia 2018^(51)^	Investigar a confiabilidade com que profissionais de saúde com diferentes níveis de especialização são capazes de transmitir a localização exata do câncer de próstata.	9 S	PMC	P
Estados Unidos da América 2018^(52)^	Avaliar o custo de joelhos controlados por microprocessador comparado aos não controlados.	15 S	EQS	AI
México 2018^(53)^	Definir o lugar da cirurgia metabólica no tratamento do DM2 no México.	24 S	PMC	AI
Austrália 2018^(54)^	Realizar testes de usabilidade do aplicativo para avaliar a dor em adultos com demência tratados por paramédicos; avaliar a adequação do algoritmo no aplicativo como fluxograma de avaliação da dor para uso em adultos com demência; identificar áreas para desenvolvimento adicional no aplicativo de dor para melhorar a interação do usuário; responder às recomendações feitas pelas equipes clínicas e trabalhar com especialistas em ciências da computação para as modificações necessárias.	7 S	DD	P
México 2019^(55)^	Descrever o *design* e o desenvolvimento de um sistema baseado na *web*.	10 S	DD	AI
Brasil 2019^(56)^	Validar o treinamento *online* para multiplicadores do programa VAMOS.	6 S	DD	P
Brasil 2019^(57)^	Validar a tecnologia educacional “Manual de cuidados familiares com a pessoa idosa submetida à cirurgia cerebral”.	11 S e OA	DND	AI
Brasil 2019^(58)^	Desenvolver e validar o conteúdo de um *software* gratuito baseado na *web* (*desktop* e mobile aplicações) para autogestão e exercícios personalizados de pé-tornozelo para pessoas com diabetes e neuropatia diabética.	3 S	DD	AI
Brasil 2019^(59)^	Elaborar e validar conteúdo e aparência de jogo educativo sobre hábitos de vida saudáveis para adolescentes.	15 S	DND	ADO
Nova Zelândia 2019^(60)^	Investigar a utilidade percebida de diferentes atividades de cuidados diários para o robô, desenvolvido a partir de pesquisas anteriores sobre necessidades.	19 S	DD	AI
Suécia 2019^(61)^	Desenvolver e avaliar um protótipo para monitorar movimentos e frequência cardíaca.	SI S e OA	EQS	AI
Índia 2019^(62)^	Desenvolver um aplicativo móvel educacional bilíngue baseado em *smartphone* para pacientes cardíacos e teste piloto em um ambiente clínico indiano.	5 S e OA	DD	AI
Brasil 2019^(63)^	Desenvolver e validar o conteúdo de um *software* gratuito baseado na *web* (*desktop* e mobile aplicações) para autogestão e exercícios personalizados de pé-tornozelo para pessoas com diabetes e neuropatia diabética.	19 S	DD	AI
Brasil 2020^(64)^	Validar o conteúdo da tecnologia educacional “Saúde do Homem: dicas para uma vida saudável”.	9 S	DND	AI
Brasil 2020^(65)^	Validar a adequação do material e usabilidade do treinamento *online* para capacitar profissionais de saúde para implementação do programa VAMOS, versão 3.0.	22 S e OA	DD	P
Brasil 2020^(66)^	Desenvolver uma intervenção educativa por meio de um jogo com abordagem de aspectos relacionados à maternidade na adolescência e cuidado da criança.	8 S e OA	DND	ADO
Estados Unidos da América 2020^(67)^	Descrever o desenvolvimento e o conteúdo de apresentações de slides e avaliar sua capacidade de suscitar discussões entre os participantes dos grupos focais.	17 S e OA	DND	AI
Estados Unidos da América 2020^(68)^	Desenvolver a intervenção COPE-STAR usando uma plataforma baseada na *web* para ajudar cuidadores de crianças com idade 1-5 anos para controlar os sintomas e a tecnologia médica de seus filhos em casa.	8 S	DD	AI
Brasil 2020^(69)^	Validar por conteúdo e usabilidade o aplicativo “NASF em Rede” direcionado às equipes dos Núcleos Ampliados de Saúde da Família e Atenção Básica.	18 S e OA	DD	P
Brasil 2020^(70)^	Avaliar a qualidade técnica de aplicativo móvel para apoiar a decisão do enfermeiro nos cuidados de prevenção de lesões de pele em recém-nascidos hospitalizados, de acordo com o Modelo de Qualidade do Produto.	20 S e OA	DD	P
Reino Unido 2020^(71)^	Desenvolver indicadores registrados pelo paciente para dispositivos de terapia manual em epidermólise bolhosa e seu uso em estudo de prova de conceito N-de-1 com análise de custos para investigar desempenho da luva de curativo descartável, comparado a curativos e bandagens convencionais.	8 S	DND	P
Portugal 2020^(72)^	Descrever e relatar os passos iniciais no desenvolvimento de um novo dispositivo médico (a seringa Duo).	9 S e OA	DND	P
Espanha 2021^(73)^	Avaliar a maturidade do sistema de classificação DESDE-AND.	23 OA	SG	P
Reino Unido 2021^(74)^	Avaliar, na opinião de anestesiologistas regionais especialistas, o desempenho geral de um sistema de Inteligência Artificial para guiar um ultrassom para confirmação da visão correta do profissional com menos experiência.	3 S	DD	AI
Índia 2021^(75)^	Avaliar a relação custo-eficácia do eletrocardiograma portátil para triagem de doenças cardiovasculares entre adultos sintomáticos e de alto risco no *Primary Health Center*.	3 S	EQS	AI
Brasil 2021^(76)^	Construir e validar uma tecnologia educacional de autocuidado para cuidadores informais.	7 S	DND	AI
Taiwan 2021^(77)^	Avaliar o custo-utilidade do Avelumabe em Taiwan.	3 S	EQS	AI
Brasil 2021^(78)^	Verificar validade conteúdo do aplicativo móvel *Safe Heart* para monitoramento e identificação de risco de infarto.	10 S e OA	DD	AI
Canadá 2021^(79)^	Criar um *kit* de ferramentas para clínicos realizarem avaliações e reabilitação em uma plataforma habilitada com tecnologias imersivas.	SI OA	DD	AI e P
Áustria 2021^(80)^	Obter conhecimento sobre a captação, aceitação do usuário e preocupações em relação a um aplicativo de predição baseado em *machine learning* projetado para melhorar a segurança do paciente em um ambiente clínico.	15 S e OA	DD	AI
Emirados Árabes 2022^(81)^	Realizar ATS para um nível diferente de intervenção de telemedicina no manejo do diabetes.	12 S	PMC	AI
França 2022^(82)^	Destacar os tipos de impactos organizacionais de impressão 3D em hospital.	12 S e OA	EQS	P
Israel 2022^(83)^	Analisar o impacto de duas tecnologias que fornecem MEDs diretamente ao cérebro.	8 S	DD	P
Tailândia 2022^(84)^	Sintetizar documentos e pesquisas internacionais de aprendizagem digital inteligente para melhorar a alfabetização digital em saúde; projetar uma aprendizagem digital inteligente; e avaliar a adequação.	10 S e OA	DD	AI
Brasil 2022^(85)^	Apresentar versão inicial do desenvolvimento tecnológico e avaliação de aplicativo que auxilia a coleta de dados, análise, avaliação e monitoramento de cantinas escolares saudáveis.	3 S	DD	RNC, ADO
Brasil 2022^(86)^	Produzir e validar tecnologia educacional em saúde em vídeo para incentivo ao aleitamento materno com famílias.	20 S	DND	RNC
Brasil 2022^(87)^	Apresentar o desenvolvimento, validação e avaliação de usabilidade de cartilha que direciona o treinamento e personaliza a progressão de programa domiciliar de treinamento de pé-tornozelo exercícios.	8 S e OA	DND	AI
Paquistão 2022^(88)^	Desenvolver uma sala de aula virtual assíncrona para ensino de fatores de testes laboratoriais.	17 OA	DD	EST
Estados Unidos da América 2022^(89)^	Desenvolver um simulador de endoscopia bariátrica virtual para treinamento e avaliação do *endoscopic sleeve gastroplasty.*	5 S	EQS	P
Itália 2022^(90)^	Avaliar os benefícios potenciais do *Magnetic Resonance-guided high intensity Focused Ultrasound*.	11 S	EQS	AI
Brasil 2022^(91)^	Avaliar a usabilidade do aplicativo “Meu PICC” para o acompanhamento extra-hospitalar de pacientes em uso de cateter central de inserção periférica.	18 S e OA	DD	AI
Brasil 2023^(92)^	Construir e validar aplicativo que estabeleça grau de risco do pé de pacientes com DM associado a condições de saúde.	18 S	DD	P
França 2023^(93)^	Analisar custo-efetividade do blinatumomab *versus* HC3 em crianças com primeira recidiva de leucemia linfoblástica aguda de alto risco.	SI S	MED	RNC
Estados Unidos da América 2023^(94)^	Avaliar a aceitabilidade e viabilidade de uma plataforma orientada para a tecnologia para facilitar a plataforma de coordenação de cuidados: Care4AD.	35 S	DD	AI
Itália 2023^(95)^	Avaliar a relação custo-benefício de radiafrequência ablação para câncer de esôfago na Itália.	5 S	EQS	AI
Itália 2023^(96)^	Avalia os benefícios e o valor acrescentado da introdução da trifluridina/tipiracilo na prática clínica italiana, definindo o comparativo e os perfis de eficácia e segurança em relação às outras opções de tratamento.	8 S	MED	AI
Suécia 2023^(97)^	Avaliar a usabilidade do protótipo *Electronic Health Report Form* para identificar saúde e problemas relacionados à saúde em jovens.	14 S e OA	DD	ADO
Portugal 2023^(98)^	Avaliar protótipo de roupa que incorpora sensores para avaliação de pressão, temperatura e umidade para prevenção de lesões por pressão.	9 S	DND	P
Colômbia 2023^(99)^	Determinar a classificação terapêutica do caplacizumabe segundo a metodologia do Instituto de Avaliação de Tecnologia em Saúde.	5 S	MED	AI
Itália 2023^(100)^	Avaliar os custos do *Radiofrequency Echographic Multi Spectrometry versus* tecnologia ionizante convencional para diagnóstico da osteoporose na perspectiva do Serviço Nacional de Saúde italiano.	6 S	PMC	AI

*S - saúde; OA - outras áreas; MED - medicamento; EQS - equipamento e suprimento; PMC - procedimento médico cirúrgico; SG - sistema de gestão; DND - dispositivo não digital; DD - dispositivo digital; RNC - recém-nascido/criança; ADO - adolescente; AI - adulto/idoso; SI - sem informação; ATS - avaliação de tecnologias em saúde; DM - diabetes mellitus; P - profissionais; EST - estudantes.*

Quanto aos países, foram identificados 44 nas Américas (27 no Brasil) e 62 (69,7%) distribuídos em 30 países, abarcando quatro continentes (Figura 2). Além disso, 46 (51,7%) foram publicados nos últimos cinco anos (2019-2023). Em relação às tipologias de tecnologias avaliadas, conforme as categorias teóricas^(1)^, foram mapeados 29 (32,6%) DDs, 19 (21,3%) PMCs, dez (11,2%) SGs, nove (10,2%) EQSs, cinco (5,7%) MEDs. Ainda, conforme a categoria empírica que emergiu, 17 (19%) dispositivos não digitais (DNDs).

**Figura 2:** Caracterização da distribuição geográfica das produções científicas com participação de especialistas na avaliação de tecnologias em saúde no período de 2013-2023

A população total de especialistas em todos os estudos incluídos foi de 1.068. Em relação ao público-alvo das tecnologias avaliadas, 52 (58,4%) foram adultos e idosos; 26 (29,2%) foram profissionais; três (3,4%) foram estudantes; três (3,4%) foram adolescentes; dois (2,2%) foram recém-nascidos e crianças; dois (2,2%) foram crianças e adolescentes; e um (1,2%) foi adulto e idoso, e profissional.

O mapeamento da participação de especialistas indicou que as características do processo de ATS estão relacionadas às tipologias das tecnologias que modificam as variáveis analisadas, como atributo avaliado, contexto em que se realizou a ATS, delineamento do estudo e técnica de participação (Figura 3).

**Figura 3:** Características da avaliação de tecnologia em saúde com a participação do especialista segundo as tipologias das tecnologias

No que tange à área de *expertise* dos especialistas, em 57 (64%) dos estudos, os especialistas eram da saúde. Somente na avaliação de MED todos eram da área da saúde.

Na avaliação de EQS e de PMC, majoritariamente (77,8% e 78,9%, respectivamente), os especialistas são da área da saúde. Quando houve especialistas de outras áreas, incluíram-se membros de comissões de ATS^(12,42,82)^. Na avaliação de EQS, incluíram-se ainda participantes da engenharia^(61)^, e na avaliação de PMC, incluíram-se participantes da área de custos exclusivamente^(36)^ e integrado à área da saúde^(45)^. Em relação aos DDs e aos DNDs, a maioria também incluiu especialistas da saúde (55,2% e 64,7%, respectivamente). Na avaliação de DD, houve participação exclusiva de especialistas de informática^(30,32,79,88)^ ou informática integrada a especialistas da saúde^(62,65,69,70,78,80,84,91,97)^. Já na avaliação de DND, ao incluir outras áreas, considerou-se a área de *design* ^(49,87)^ ou associou-se aos participantes da área da saúde outros especialistas de gestão^(66,67)^, pedagogia^(57)^ e informática^(72)^. Na avaliação de SG, a participação de especialistas da área da saúde não foi maioria (40%). Quando houve especialistas de outras áreas, incluíram-se a área de gestão da informação^(27)^, informática exclusivamente^(73)^ e informática integrada à área da saúde^(26,28,37,40)^.

No que se refere à participação deste especialista, o número de especialistas por estudo variou de três a 158. A amostra foi composta de acordo com a tipologia de tecnologia avaliada. Verificou-se que o mínimo de especialistas foi de três na avaliação de EQS^(75,77)^, DD^(34,58,74,85)^, DND^(16)^ e PMC^(42)^, e cinco, na avaliação de MED^(31)^ e SG^(27)^. Os números máximos se aproximaram de 30 na avaliação de MED^(31)^, DD^(94)^ e SG^(26)^, e diminuíram para 20, na avaliação de DND^(86)^, e para 15, na avaliação de EQS^(52)^. Na avaliação de PMC, houve um único estudo^(33)^ que atingiu um máximo de 158.

Em 11 estudos, não foram encontradas informações sobre o quantitativo de especialistas^(13,15,25,29,35-38,61,79,93)^. Não foram identificadas citações para justificar o quantitativo, seja mínimo ou máximo. Considerando a amostra (n=89), verificamos que o mínimo de três e o máximo de 15 ocorreram em 64% dos estudos.

Em todas as tipologias, a seleção se deu em instituições de referência nacionais e internacionais. A menção à Plataforma *Lattes* como única fonte para seleção dos especialistas só foi identificada nos estudos do Brasil na avaliação de DND^(76,86,87)^ e DD^(69,70,78)^, sendo integrada a seleção em instituições na avaliação de DND^(66)^. Houve menção à seleção somente na literatura para avaliação de EQS^(52,82)^ e SG^(21)^, integrada a instituições na avaliação de PMC^(41)^. Entretanto, em todas as tipologias (80% na avaliação de MED; 68% na avaliação de PMC; 66% na avaliação de DD; 65% na avaliação de DND; 60% na avaliação de SG; 56% na avaliação de EQS), não foi encontrada informação.

Sobre os atributos avaliados, destaca-se o atributo propriedade, predominante em DD e DND (93% e 88%, respectivamente), seguido de SG (50%), EQS (33,3%) e PMC (10%), sendo que, para MED, não se avaliou a propriedade. A efetividade foi avaliada isoladamente em MED^(96)^, EQS^(95)^, DD^(83)^ e PMC^(23,24,100)^, e integrada a custos em MED^(19,31,93)^ e EQS^(44,75)^. A utilidade se destacou em PMC^(13,14,35,41,47,53)^, seguida de SG^(37,45)^, MED^(99)^, DD^(94)^ e DND^(15)^. A avaliação de custos foi realizada isoladamente na avaliação de PMC^(36,38,45)^ e EQS^(52)^, sendo integrada à utilidade^(77)^, segurança^(72,81)^ e impacto^(39)^ na avaliação de EQS e PMC. A avaliação da segurança se destacou na avaliação de SG^(20,21)^. A avaliação de impacto^(82)^ e riscos^(12)^ foi realizada em EQS e PMC.

Em relação ao contexto em que se realizou a ATS, constatou-se que a modalidade *online* foi a mais utilizada na avaliação dos DNDs (53%) e DDs (52%). A modalidade presencial foi a mais utilizada na avaliação de EQS (77,7%). Destacou-se, nas tipologias PMC^(13,14,23,33)^, DND^(67,71,76)^, SG^(40,73)^, EQS^(52)^ e DD^(79)^, a modalidade mista, que utilizou tanto a modalidade *online* quanto a presencial.

Os delineamentos dos estudos de ATS em que os especialistas participaram foram majoritariamente quantitativos em todos as tipologias: MED (100%); SG (90%); EQS (77,7%); PMC (73,7%); DD (65,5%); e DND (64,7%). Os delineamentos qualitativos só não foram identificados na avaliação de MED, e os estudos de métodos mistos foram utilizados na avaliação de PMC^(24)^, DND^(72,98)^ e DD^(80,85,8897)^.

As técnicas mais utilizadas para participação dos especialistas foram os instrumentos estruturados (questionários e formulários), atingindo 48% em estudos de avaliação de DD, 47%, de DND, e 36%, de PMC. Na avaliação de DD, a diversidade de técnicas combina o questionário com pensamento em voz alta^(29,30)^, utilização de aplicativo^(85)^, grupo focal isoladamente^(43,80)^ e combinado com testagem e painel Delphi^(53)^, exposição seguida de manuseio e instrumento de avaliação^(32)^, além de entrevista após apresentação de tecnologia^(60)^, manuseio do protótipo e pontuação^(62)^, utilização do software e testes a partir de estudos de caso^(70)^ e simulação^(79)^.

Na avaliação de SG^(20,21,28,37)^ e DD^(16)^, destacou-se a utilização de entrevistas isoladamente. Na avaliação de EQS, utilizou-se a entrevista combinada com grupo focal^(82)^, questionário^(90)^ e painel de discussão por telefone^(52)^. Também em estudos de avaliação de EQS, outras técnicas combinadas foram identificadas, como reuniões regulares, grupo focal, *workshop* ^(61)^, utilização de modelos e questionário^(77)^, filmagem de procedimento, e questionário^(89)^.

Na avaliação de MED, destacou-se a utilização de Delphi modificado^(31,99)^. Na avaliação de DND e PMC, também se verificou a utilização de técnicas isoladas como grupo focal^(12,17,72,81)^, Delphi modificado^(33)^ e entrevista^(23,24,38,53,100)^. Na avaliação de DND, foram utilizadas técnicas combinadas, como reuniões e *design* colaborativo, com discussões presenciais e reuniões por telefone^(67)^, reunião, troca de e-mails^(71)^, questionário e grupo focal^(98)^. Na avaliação de PMC, foram utilizados análise de estudo de caso e grupo focal^(13)^, entrevista, questionário^(14)^, plotagem e desenhos^(51)^.

## DISCUSSÃO

As evidências disponíveis mapeadas nesta revisão de escopo mostraram que a participação dos especialistas nos processos de ATS tem caraterísticas de acordo com as tipologias de tecnologias em saúde avaliadas. Tais características se modificam em cada tipologia avaliada no que tange à área de *expertise*, atributo avaliado, seleção, amostra, contexto, delineamento e técnica.

Uma das características é que, na ATS, há participação de uma variedade de especialidades, e existem diferentes concepções sobre quem é considerado um especialista. Destacam-se nos processos avaliativos os especialistas em conteúdo que têm conhecimento sobre a temática da tecnologia, que podem vir de diferentes áreas de especialização, mas principalmente da área da saúde, bem como os especialistas em métodos que irão contribuir com a garantia de qualidade^(8)^. O mapeamento apontou para participação de especialistas de outras áreas além da saúde, a exemplo da engenharia, da informática, do *designer*, da pedagogia, entre outras. Uma recomendação para futuros estudos é mapear desafios por áreas, inclusive a da enfermagem.

Outra característica é a variedade de estratégias. Os estudos realizaram avaliações de diferentes tipos de dispositivos, como digitais, não digitais e procedimentais, o que converge com recomendações de pesquisadores da China, que afirmam que os dispositivos diferem consideravelmente dos produtos farmacêuticos em muitos aspectos, o que torna a ATS desafiante. Os autores fazem um apelo tanto às comunidades acadêmicas quanto às agências, para que se padronizem o processo, metodologias e critérios de ATS sobre dispositivos, com vistas a ampliar as avaliações, pois as atuais diretrizes de ATS ainda se centram principalmente nos MEDs^(101)^.

Apesar do desafio dos processos de ATS, a variedade de especialidades e de estratégias favorece a obtenção de diferentes análises e contribuições, o que aponta para a triangulação das áreas dos participantes, de delineamentos e de técnicas. A triangulação é uma estratégia de aprimoramento dos estudos, envolvendo diferentes perspectivas, utilizada para aumentar a sua credibilidade, ao implicar a utilização de dois ou mais aspectos e possibilitar a apreensão do fenômeno avaliativo sob diferentes níveis, considerando a complexidade dos objetos de estudo (as tecnologias em saúde) e condições do próprio processo avaliativo^(102)^.

Nesse processo, quanto às áreas de *expertise* dos especialistas, ficou evidente que há estudos só com especialistas da área da saúde, estudos em que participantes da área da saúde se integram a diferentes áreas, e estudos em que outras áreas participam de modo isolado. Também se verificou que, em todas as tipologias de tecnologias avaliadas, houve participação de especialistas da área da saúde, com destaque para a tipologia MED, em que a saúde foi a única especialidade em todos os estudos.

Os especialistas da área da saúde participaram, de modo isolado ou de forma integrada com outras áreas, majoritariamente na ATS das tecnologias, com exceção de SG. A área do especialista a ser integrada à saúde depende da tipologia da tecnologia avaliada. Entre os países da União Europeia, Reino Unido, Canadá e Austrália, encontram-se especialistas da área da saúde e de outras áreas participam dos estudos de ATS. A participação de especialistas de diferentes áreas pode garantir a transparência e inclusão de reflexões de diferentes perspectivas^(103)^.

Em estudo onde especialistas forneceram estimativas para questões técnicas, tanto na área de especialização em que eram referência quanto nas áreas em que não eram, descobriu-se que os participantes eram mais sérios e cautelosos em relação às tarefas nos estudos relacionados diretamente às suas especialidades. Logo, a área de *expertise* do especialista influencia a ATS. A recomendação é que, para qualquer processo de ATS, todos os especialistas tenham a experiência substantiva necessária e um conhecimento relevante sobre o tópico em avaliação^(104)^.

Sobre a amostra nos estudos de ATS, foi possível mapear a quantidade de especialistas, bem como verificar o número mínimo e máximo, o que apontou para uma variação entre três e 15. Ademais, não foram encontradas menções nos estudos a padrões ou modelos que determinam o quantitativo de especialistas a ser atingido, o que reforça a assertiva de que não há padrão estabelecido acerca do quantitativo de especialistas^(105)^.

Em estudo que considerou 22 organizações de ATS e incluiu 42 documentos, sobre a amostra de especialistas, verificou-se que a participação, seja de modo individual ou grupal, era com números variados de membros. Algumas agências recomendam a inclusão de mais de um especialista para obter uma gama de pontos de vista. Ressalta-se que o tamanho dos painéis de especialistas indicados na diretriz da Irlanda varia entre cinco e 20 indivíduos^(8)^, o que converge com o mapeamento realizado nesta revisão.

Ao mapear as evidências sobre a seleção dos especialistas, verificou-se um elevado número de estudos em que não foram encontradas informações sobre as fontes consultadas para seleção dos especialistas, atingindo-se o maior percentual nos estudos de avaliação de MED (80%). Compreendese que a seleção é uma tarefa desafiadora para os estudos de avaliação, devendo os pesquisadores estabelecer critérios quantitativos e qualitativos, almejando resultados confiáveis. Ressalta-se, sobre a seleção dos especialistas, que a análise acurada das características de uma tecnologia está relacionada à confiança e experiência que o especialista possui na área, devendo ser capaz de analisar, interpretar e inferir valores quantitativos e/ou qualitativos^(105)^.

Quanto ao mapeamento das evidências acerca do atributo avaliado pelos especialistas, foram identificados estudos em que propriedades foram avaliadas (conteúdo, aparência, usabilidade, qualidade), o que caracteriza uma avaliação no primeiro ciclo de vida da tecnologia, antes do registro, quando são verificados aspectos técnicos e operacionais da tecnologia. Nesses estudos, quando a propriedade avaliada foi conteúdo e aparência, majoritariamente foi utilizado o termo “validação”, enquanto nos estudos em que a propriedade avaliada foi usabilidade e qualidade, o termo foi “avaliação”.

Quanto aos atributos voltados aos aspectos de resultados, mais característicos do segundo ciclo de vida em diante, foram encontrados estudos de avaliação de efetividade, utilidade, custos, segurança, impacto e riscos. Considera-se relevante que na ATS se realizam estudos em diferentes pontos do ciclo de vida de uma tecnologia em saúde^(106)^, pois o processo é caracterizado por ser sistemático e contemplar tanto as propriedades quanto os efeitos e/ou impactos, com vistas a melhorar a qualidade e o valor da tecnologia, facilitando, assim, decisões^(103)^.

Historicamente, a ATS tem se centrado na segurança e eficácia de uma intervenção e também na relação custo-eficácia, embora haja uma consciência crescente de que é necessária uma forma mais ampla de ATS. Ressalta-se que já estão em curso alguns esforços, mas limitados a poucos países, para atender a essa necessidade. Se a ATS deixar de expandir a sua avaliação e recomendações, sem incluir outras questões como as propriedades, por exemplo, poderá ser considerada irrelevante para as reais necessidades dos decisores. A ATS deveria (e faz cada vez mais) possuir recomendações sobre tecnologias que vão além da segurança, eficácia e custo-benefício^(107)^, o que, no rastreamento realizado, ficou evidente. A segurança, um dos atributos identificados no mapeamento, com destaque para os estudos de avaliação de SG, em que foi avaliada isoladamente^(20)^ e integrada a riscos^(21)^, busca garantir que as tecnologias em saúde sejam analisadas para, assim, minimizar os riscos para os pacientes e otimizar os resultados desejados^(108)^.

Sobre o contexto, que se refere aos ambientes que foram utilizados para que os especialistas pudessem interagir com as equipes de pesquisa (*online* ou presencial), as evidências convergiram para uma perspectiva multidimensional, pois, nas tipologias DND e DD, houve predominância do modo *online*, e na tipologia EQS, a predominância foi da modalidade presencial. Outra evidência é que houve a utilização dos dois contextos em estudos que avaliaram as tipologias PMC^(13,14,23,33)^, DND^(68,71,76)^, SG^(40,73)^, EQS^(52)^ e DD^(79)^.

Entendemos que os dois contextos têm pontos positivos e negativos. Autores reconhecem que conduzir uma consulta a um especialista de modo presencial requer um esforço significativo, acesso a recursos, treinamento e tempo, especialmente quando vários especialistas estão envolvidos, como nos estudos de ATS. Pode ser muito demorado, especialmente em áreas de doenças onde há poucos especialistas e muitos estão geograficamente dispersos e com agendas lotadas. Além disso, durante a consulta presencial, o facilitador precisa ter cuidado para não impor suas crenças ou preconceitos ao especialista. Com a utilização do ambiente *online*, mais especialistas podem ser alcançados em um tempo mais curto, porém, geralmente, o custo de taxas de resposta muito baixas, especialmente quando o alvo são médicos, tende a reduzir os custos de levantamento de dados e o nível de erros de registro^(104,109)^.

Em relação aos delineamentos dos estudos em que participaram os especialistas, somente na avaliação de MED não foi utilizada a avaliação qualitativa. Nos demais, além de se ter estudos quantitativos e qualitativos, também foram identificados estudos de métodos mistos na avaliação de PMC^(24)^, DND^(72,98)^ e DD^(80,85,88,97)^.

A opinião de especialistas pode ser qualitativa, usada para validar os caminhos de saúde simulado em um modelo de custo-efetividade, mas também quantitativa, usada para fornecer estimativas, por exemplo. A consulta mais estruturada visa facilitar o processo de obtenção de dados quantitativos com vistas a se ter opiniões tão rigorosas e científicas quanto possível. Esses métodos permitem que os especialistas especifiquem uma quantidade de interesse, bem como a incerteza associada em torno dela, que pode ser codificada como uma distribuição de probabilidade^(110)^. O delinemento da ATS majoritariamente quantitativo em todas as tipologias de tecnologias é coerente com o atributo avaliado. Por exemplo, na tipologia MED, o atributo majoritariamente avaliado foi custo-efetividade, que requer um delineamento quantitativo. Outro exemplo são os delineamentos quantitativos nas avaliações de propriedade, em que foram mapeados principalmente o uso de cálculo de Índice de Validade de Conteúdo.

Sobre as técnicas que foram aplicadas com os especialistas, destacou-se a diversidade de modalidades, tanto aplicadas isoladamente quanto combinadas. Em todas as tipologias, utilizaram-se tanto questionários e formulários quanto grupos focais, entrevistas e o método Delphi, o que é recomendado para coletar as opiniões dos especialistas^(8)^. Especificamente sobre o método Delphi, há inúmeras características que são consideradas vantajosas como: o anonimato entre os participantes, que evita constrangimentos, inibições e intimidações que podem ocorrer em encontros presenciais, possibilitando a expressão de comentários de todos, mesmo daqueles especialistas cujas opiniões são minoritárias; a possibilidade de fornecer aos participantes *feedback* por suas contribuições; a possibilidade de revisão das respostas dos especialistas, bem como a formação de grupos heterogêneos, quando o assunto ou produto a ser avaliado for multidimensional e multidisciplinar. Quando é aplicada em meio virtual, há outro diferencial e vantagem para sua utilização, pois permite maior tempo ao participante para reflexão em relação à tecnologia avaliada, podendo levar à maior adesão, participação, além de menor custo e tempo despendido para sua realização^(105,111)^.

Acreditamos ser importante adotar distintas técnicas nos estudos de ATS, pois permitem o levantamento de perspectivas tanto objetivas quanto subjetivas. A opinião obtida por meio de entrevistas individuais (realizadas pessoalmente ou remotamente) ou por meio de entrevistas escritas com respostas a perguntas específicas favorece a análise de diferentes perspectivas na manifestação dos especialistas^(110)^. No que tange aos questionários, em relação ao contexto em que são aplicados, ressalta-se que, no âmbito *online*, apresentam maior retorno do que os autopreenchidos de modo presencial, permitindo alcançar uma área geográfica mais ampla; outra vantagem é que não existe pressão pela presença do pesquisador, pois o anonimato pode ser preservado plenamente, e existe um custo menor do que a realização, por exemplo, de uma entrevista ou grupo focal^(109,112,113)^.

Os grupos focais estimulam as trocas de vivências, experiências e impressões, o que pode possibilitar reflexão coletiva sobre a tecnologia que se está avaliando. A técnica proporciona a configuração de um ambiente que permite a problematização e o aprofundamento de discussões, permitindo, acima de tudo, o conhecimento e a compreensão das distintas *expertises* dos sujeitos. As questões levantadas sobre a tecnologia em pauta podem provocar reflexões a respeito do processo de avaliação em si e suscitar novos significados da própria experiência avaliativa^(114,115)^.

Esse resultado apontou uma lacuna nos estudos de ATS no que se refere à falta e/ou incompletude de informações que caracterizam a participação de especialistas no processo de ATS, em especial no que se refere à seleção, contexto e técnica utilizada.

### Limitações do estudo

A limitação desta revisão foi o não desenvolvimento da terceira etapa da busca e seleção das evidências, que se refere à busca manual na lista de referências dos estudos incluídos na amostra.

### Contribuições para a área da enfermagem

A revisão contribui para o campo da saúde, ao oferecer uma análise abrangente da participação de especialistas na avaliação de diversas tecnologias de saúde. Essas descobertas enfatizam a necessidade de se delinear de forma mais estruturada a avaliação de acordo com as características específicas da tipologia de tecnologia, fornecendo *insights* para implementação de inovações nesse setor. Dessa forma, a revisão não apenas amplifica o conhecimento sobre a participação de especialistas, mas orienta futuras pesquisas e práticas na área da saúde.

## CONCLUSÕES

O mapeamento das evidências sobre a participação de especialistas no processo de ATS mostrou que o atributo avaliado é uma das características definidoras da participação do especialista e está diretamente relacionado ao tipo de tecnologia. A área de *expertise* predominante para inclusão na avaliação é a da saúde, com exceção das tecnologias classificadas como SG, e a inclusão de outras áreas depende do tipo de tecnologias em avaliação. A amostra de especialistas variou, na maioria dos estudos, de três a quinze. A seleção dos especialistas se dá por meio de informações obtidas em instituições e na literatura, mas esta informação é uma lacuna, pois há pouca transparência nos estudos deste dado.

## Data Availability

Os dados de pesquisa estão disponíveis no corpo do artigo.
